# The genetic profile and molecular subtypes of human pseudomyxoma peritonei and appendiceal mucinous neoplasms: a systematic review

**DOI:** 10.1007/s10555-023-10088-0

**Published:** 2023-02-01

**Authors:** Nora Wangari Murage, Nada Mabrouk Ahmed, Timothy J. Underwood, Zoë S. Walters, Stella Panagio Breininger

**Affiliations:** 1 School of Cancer Sciences, Faculty of Medicine, University of Southampton, Southampton, SO17 1BJ UK; 2 Pathology Department, Faculty of Medicine, Alexandria University, Alexandria, Egypt; 3 Institute of Cardiovascular Sciences, University College London, London, UK

**Keywords:** Pseudomyxoma peritonei, Appendiceal mucinous neoplasms, Somatic gene mutations, *KRAS*, *GNAS*, Survival

## Abstract

Pseudomyxoma peritonei (PMP) is a rare, progressive, slowly growing neoplastic condition which is poorly understood, with a 5-year progression-free survival rate as low as 48%. PMP is most commonly caused by appendiceal mucinous neoplasms (AMN), and understanding their genetic biology and pathogenicity may allow for the development of better novel systemic treatments to target key deleterious mutations and the implicated pathways. The primary aim of this systematic review was to identify the genetic profile of histologically confirmed human PMP or AMN samples. The secondary aim was to identify whether genetic marks could be used to predict patient survival. Ovid EMBASE, Ovid MEDLINE, PubMed, and Web of Science were searched to identify studies investigating the genetic profile of histologically-confirmed human PMP or AMN samples. We review findings of 46 studies totalling 2181 tumour samples. The most frequently identified somatic gene mutations in patients with PMP included *KRAS* (38–100%), *GNAS* (17–100%), and *TP53* (5–23%); however, there were conflicting results of their effect on survival. Three studies identified molecular subtypes based on gene expression profiles classifying patients into oncogene-enriched, immune-enriched, and mixed molecular subtypes with prognostic value. This review summarises the current literature surrounding genetic aberrations in PMP and AMNs and their potential utility for targeted therapy. Given the recent advances in clinical trials to directly target *KRAS* and *GNAS* mutations in other cancers, we propose a rationale to explore these mutations in future pre-clinical studies in PMP with a view for a future clinical trial.

**Graphical Abstract:**

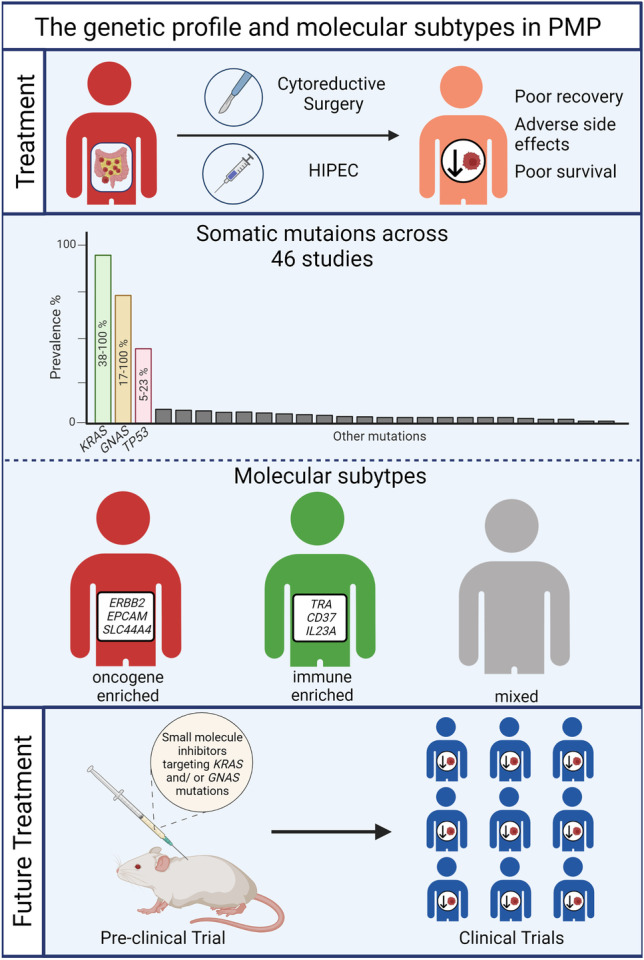

**Supplementary Information:**

The online version contains supplementary material available at 10.1007/s10555-023-10088-0.

## Introduction

### The prevalence and development of pseudomyxoma peritonei

**Fig. 1:**
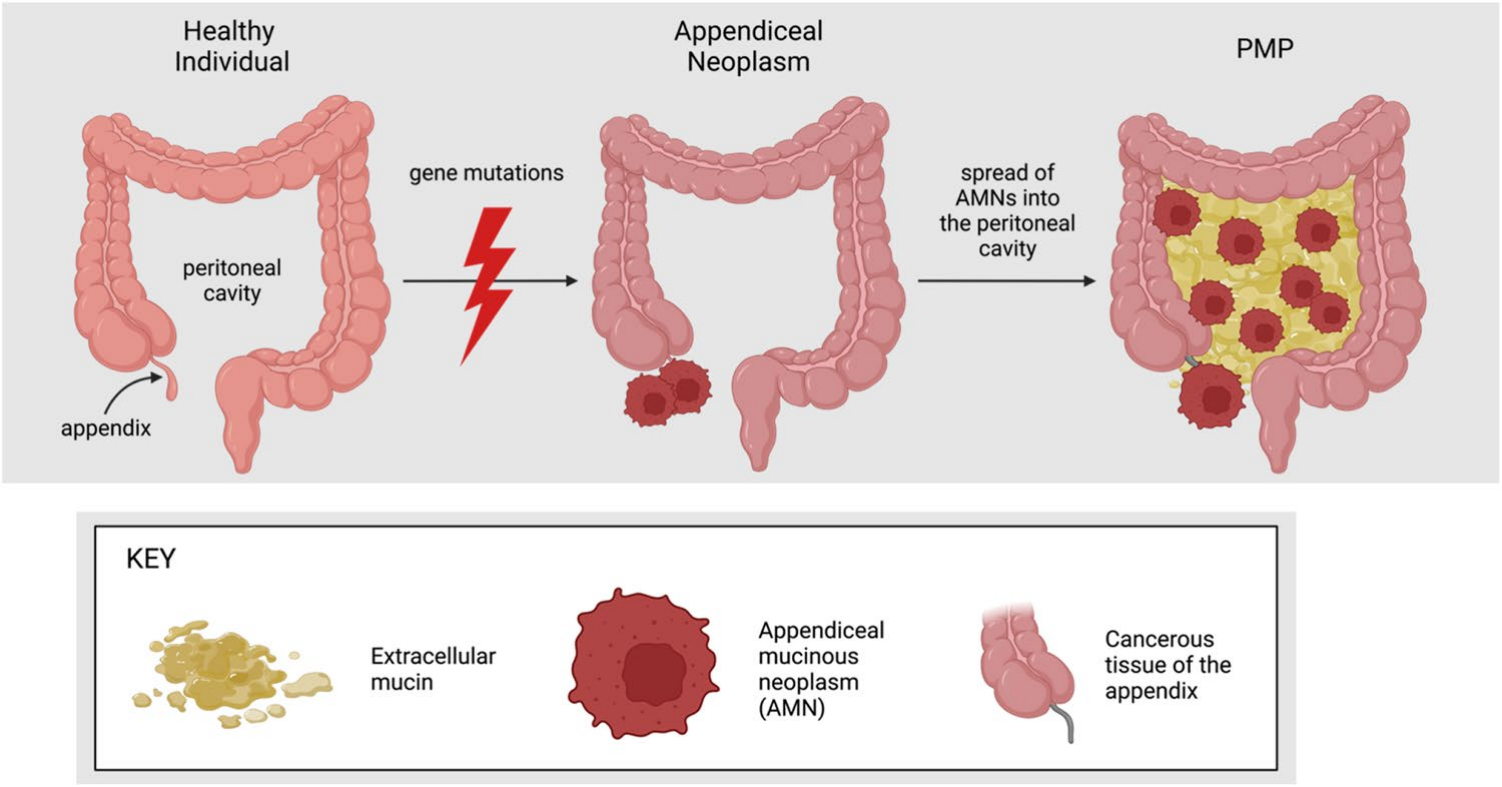
The physiological sequelae of PMP. AMN and extracellular mucin accumulate in the appendix causing it to rupture. These neoplasms will metastasise throughout (and are confined to) the abdominopelvic cavity following the redistribution phenomenon. Consequently, the patient will develop mucinous ascites, peritoneal implants, and abdominal pain; and there may be metastasis to the ovaries in female patients. (Created with BioRender.com, accessed on 4 September 2022)

Pseudomyxoma peritonei (PMP) is a rare malignant clinical syndrome with an estimated incidence of 1–2 per 1,000,000 [1]. Appendiceal mucinous neoplasms (AMN) are a common type of appendiceal tumour where the tumour cells and extracellular mucin may accumulate causing the appendix to rupture. This results in the dissemination and metastasis of the AMN and mucin into the peritoneal cavity and specific anatomical sites (including the greater omentum, under-surface of the right hemidiaphragm, right subhepatic space, and paracolic gutters) [2, 3] following the redistribution phenomenon, which leads to the development of PMP (Fig. 1) [4]. The redistribution phenomenon is a character-defining feature of PMP and occurs when extracellular mucin follows the normal flow of peritoneal fluid, redistributing the neoplastic cells [3, 5] (Fig. 1). PMP is most commonly caused by mucinous neoplasms of appendiceal origin; however, there are reported cases of ovarian, colonic, and pancreatic origin in the literature [2, 6–8]. PMP may be caused by both low-grade mucinous appendiceal neoplasms (LAMN) and high-grade mucinous appendiceal neoplasms (HAMN) [3]. Figure 1 depicts the physiological transformations that occur in PMP.

### Nomenclature of PMP and appendiceal mucinous neoplasms

Previous literature has noted various classification systems used to grade PMP and AMN, which has proven to be confusing [7, 9, 10]. To address these discrepancies, Carr et al*.* [3] and the Peritoneal Surface Oncology Group International (PSOGI) reached a consensus on the terminology (refer to Supplementary Table 1 for detailed classifications), and PMP is now classified according to the peritoneal histology instead of the primary tumour [2].

### Current treatment options for PMP

Cytoreductive surgery (CRS), where the tumour and extracellular mucin are removed, combined with hyperthermic intraperitoneal chemotherapy (HIPEC) with mitomycin-C and cisplatin is the gold standard treatment for patients with PMP [11–18]. CRS-HIPEC is currently the only treatment available with potential chances of long-term disease control and cure for these patients, as supported by a recent retrospective analysis of nearly 2000 patients [19]. Although some studies have noted a 5-year overall survival (OS) of more than 50% [14, 19, 20], another study concluded that patients have a much lower progression-free survival (PFS) rate of 48% with 31% of the sample had disease progression despite this treatment [21]. PMP patients with good prognostic factors, including low-grade histology, low peritoneal load, and no residual macroscopic disease have high chances of being cured by CRS-HIPEC [17]. Contrarily, for PMP patients with poor prognostic factors such as high grade or signet ring histology and unresectable or recurrent disease, the therapeutic options are limited, and the currently available systemic treatment options are inefficient to change the natural progression of the disease [17]. There is an unmet need for better and rational treatment options with reduced adverse side effects for these patients.

Genomics have been used to successfully guide treatment in other cancers. They are useful in understanding disease progression and identifying actionable targets for novel treatments as accomplished in colorectal [22, 23], lung [24], and breast cancers [25]. Although the genetic profile of PMP and AMN has been previously studied, currently they have not identified unique genetic targets to allow for treatments with reduced adverse side effects. This review aims to identify genetic alterations in PMP of appendiceal origin and look at survival outcomes. Collating evidence on the genetic aberrations present in PMP and AMN to date will not only identify recurrent and well-known driver genes, as well as rare gene events, but also help characterise the molecular mechanisms that define this disease [26]. Consequently, such a comprehensive characterisation can be utilised to identify established mutations with a druggable target and guide areas for future research focussing on translational drug discovery that target these genetic aberrations and mechanisms. The hope would be that this will ultimately lead to improved treatments with less adverse side effects, that are linked to the genetic profiles of PMP aiding clinicians during their decisions on treatment plans. Here, we review evidence of 46 studies that investigated genetic aberrations in PMP and AMNs samples, and we provide a comprehensive compendium of what is currently known about the genetic profile and molecular subtypes of PMP and AMN.

## Methods

**Fig. 2:**
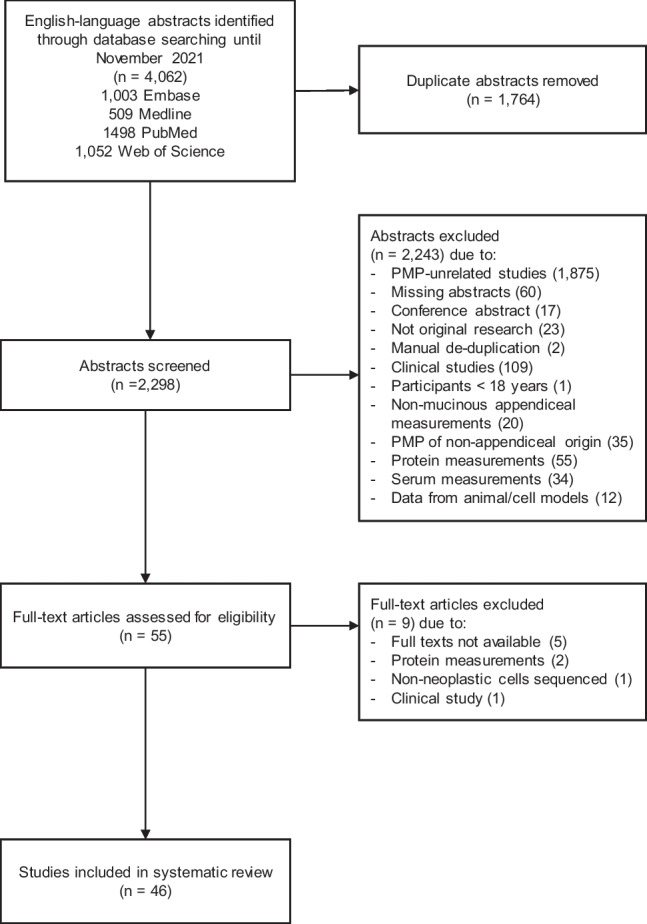
PRISMA flowchart outlining the screening process

A systematic literature search (registered on PROSPERO under the registration number CRD42021228193) was performed on four electronic databases; Ovid EMBASE, Ovid MEDLINE, PubMed, and Web of Science, to identify genetic aberrations in PMP and AMN (Table 1, Fig. 2). The search was limited to studies written in English language published between 1995 and 2021. Titles, abstracts, and full texts were screened by two independent researchers (NWM and NMA) and any disagreements resolved by a third researcher (SPB). Studies were included according to the following criteria: original research; human adults diagnosed with PMP or AMN; and studies of oncogenic marks. Studies were excluded using the following criteria: PMP of other origin other than AMN; participants under 18 years old; data from animal or cell models; and any other study not related to PMP or AMN. The reference list of a systematic review which identified somatic alterations in AMN by Stein et al. 2020 [27] was searched; however, no additional studies matching the inclusion criteria were identified. The search strategy has been outlined in Table 1 and the screening process is shown in an adapted version of the Preferred Reporting Items for Systematic Reviews and Meta-Analyses (PRISMA) flowchart in Fig. 2 [28].

**Table 1:** Search terms used for the systematic literature review search

	Search terms
1	Genetic OR genome OR exome OR molecular OR marker* OR mutation OR alteration
2	Appendi* AND mucinous AND neoplasms
3	2 OR pseudomyxoma peritonei OR PMP OR disseminated peritoneal adenomucinosis OR peritoneal mucinous carcinomatosis
4	1 AND 3

The following data items were extracted: sample size and classification of tumours; age; gender; tissue processing; experimental method used; genetic markers of human resected tissue of patients diagnosed with PMP or mucinous appendiceal neoplasms; implicated gene pathways; and survival analyses. To provide an accurate assessment of the quality of the included studies the risk of bias in non-randomised studies of interventions (ROBINS-I) tool was used which included four main domains: confounding bias, selection bias, information bias, and reporting bias (Supplementary Table 2) [29]. Studies were scored as low, moderate, or high, risk of bias.

## Results

This review included 46 studies, a total of 2181 tumour samples (sample size range across studies 1–374) of various histological subtypes of PMP and AMN: LAMN, HAMN, mucinous adenocarcinomas (MAC), mucinous cystadenocarcinomas, mucinous cystadenomas, and PMP due to low-grade neoplasms (low-grade mucinous carcinoma peritonei LGMCP) and high-grade neoplasms (high-grade mucinous carcinoma peritonei HGMCP) (Table 2). The mean and median age ranged from 50.9 to 68.0 and from 51 to 61, respectively. However, there was a wide age range of 20 to 94 across all 46 studies. Both males and females were affected; however most studies (*n*=26) had a range of 51–100% of females in their samples.

**Table 2:** Patient characteristics and key findings for studies included in this systematic review

Study	Sample size (*N*)	Age (mean years and ± SD or range)	Gender: male *N* (%), female *N* (%)	Tissue processing	Experimental method used	Key finding and implication
Flatmark et al. 2021 [32]	*N*=23 (17/23 LAMN, 3/23 HAMN, 2/23 MAC, 1/23 not classified) (20/23 low-grade PMP, 2/23 high-grade PMP, 1/23 high-grade PMP with signet ring cells)	56.6 ± 12.2 (range 32–74)	*M*= 9 (39) *F*= 14 (61)	Fresh-frozen or FFPE tumour samples	NGS with the Ion GeneStudio S5 system and the Oncomine Comprehensive Assay V.3 (Thermo Fisher Scientific) PCR (BioRad) for 10 cases	♢ Co-existing *KRAS* and *GNAS* mutations in LAMN and HAMN ♢ Co-existing *KRAS* and *GNAS* in low-grade and high-grade PMP (87%) ♢ Gs-alpha peptide vaccine may support pre-existing immunity in patients with PMP and *GNAS* mutations against Gs-alpha
Moaven et al. 2020 [66]	Total *N*=79 (17/79 Immune enriched [13 LG, 4HG], 35/79 mixed [21 LG, 14 HG], 27/79 oncogene enriched [14 LG, 13 HG])	52.3 ± 12.1	*M*= 39 (49) *F*=40 (51)	FFPE tumour blocks or fresh-frozen tumoural tissues	NanoString n-Counter Profiling technique	♢ Oncogene-enriched has significantly poorer prognosis compared to immune-enriched. ♢ *PRSS3* and *EFNA1* associated with poorer overall survival in LG. ♢ Multiple genes associated with poorer overall survival in HG (involved in cell cycle, proliferation, mucin production, adhesion, and immune pathways). ♢ 5 tumour-suppressor genes (involved in immune pathways) associated with better overall survival in HG.
Liao et al. 2020 [33]	*N*=32 (8/32 LAMN, 9/32 HAMN, 10/32 MAC)	56 range (32–83)	*M*=16 (50) *F*=16 (50)	Paraffin-embedded tissue sections	NGS using Ion AmpliSeq Cancer Hotspot Panel v2	♢ Co-existing *KRAS* and *GNAS* mutations: 63% in LAMN, 56% in HAMN, and 10% in MAC ♢ *TP53* mutations in HAMN and MAC only ♢ Progression from LAMN to HAMN might involve *TP53* and ATM mutations ♢ MAC distinct from HAMN, unique mutations
King et al. 2020 [34]	*N*=2 LGMCP from LAMN Patients are mother and daughter	53	*F*=2 (100)	FFPE tumour samples	NGS using Illumina HiSeq 4000	♢ Common pathogenic mutations in mother and daughter: *KRAS* and *RAD51C* ♢ Different *GNAS* mutations variants in mother (R201H) and daughter (R201C) ♢ Pathogenic mutation in mother only: *DIS3* ♢ Pathogenic mutation in daughter only: *RB1*
Yanai et al*.* 2020 [35]	*N*=51 (28/51 LAMN, 6/51 LAMN+PMP, 1/51 HAMN, 7/51 HAMN +PMP, 8/51 MAC, 1/51 MAC+PMP)	LAMN: 57.1 ± 13.7 (range 22–81) HAMN: 60.5 ± 13.2 (range 46–81) MAC: 63.2 ± 10.6 (range 43–72)	*M*=13 (25) *F*=38 (75)	FFPE samples, micro-dissection	NGS for 18 cases using Ion PGM^TM^ system and cancer hotspot panel v2 Sanger for remaining 31 cases using BigDye Terminator v3.1 Sanger in all cases for *RNF43* mutations Fluorescent-labelled primers for *TP53* LOH	♢ Co-existing *KRAS* and *GNAS*: 15% in LAMN, 13% in HAMN, 22% in MAC ♢ *RNF43* mutations occurred almost exclusively to *GNAS*/*KRAS* mutations in LAMN
Foster et al. 2020 [36]	*N*=13 LGMCP	65	*M*=3 (23) *F*=10 (77)	FFPE samples, manual micro-dissection	NGS using the Ion Ampliseq HotSpot Cancer Panel v2 for 50 oncogenic and tumour suppressor gene hotspots on the Ion Torrent Personal Genome Machine or Ion S5 instrument	♢ Co-existing *KRAS* and *GNAS* mutations in whole sample ♢ High rate of *TP53* mutation seen in sample of low-grade neoplasms
Lung et al. 2020 [60]	*N*=2 PMP (1 PMP due to ruptured LAMN, 1 PMP and a moderately differentiated MAC) Patients are from the same family	58.5	*M*=1 (50) *F*=1 (50)	FFPE PMP specimens, manual micro-dissection	Sanger sequencing using Big Dye Terminator v3.1	♢ Identified candidate variants that may predispose this family to PMP ♢ Shared nonsense *REEP5* mutation implicated in the regulation of *TP53* and the stabilisation of the endoplasmic reticulum
Su et al. 2020 [67]	*N*=138 appendiceal mucinous neoplasms (76/138 LG, 38/138 HG)	Whole cohort 52.0 ± 12.1 LG: 53.1 ± 13.2 HG: 50.9 ± 9.0	Whole cohort *M*=53 (43) *F*=69 (57) LG: *M*=30 (43) *F*=40 (57) HG: *M*=15 (44) *F*=19 (56)	Frozen or FFPE samples	NanoString n-Counter profiling with a 148-gene panel	♢ Subtypes may have clinical utility for predicting surgical outcomes ♢ Immune-enriched: favourable outcome, oncogene-enriched: poorer outcome, more aggressive tumour growth phenotype ♢ Oncogene-enriched, good candidate for preoperative chemotherapy trials ♢ Immune-enriched, good candidate for preoperative immunotherapy studies
Tsai et al. 2019 [37]	*N*=31 (17/31 LAMN, 6/31 LAMN+PMP, 4/31 HAMN, 1/31 HAMN +PMP, 3/31 high-grade mucinous carcinoma)	61.8	*M*=12 (39) *F*=19 (61)	FFPE samples	NGS using MiSeq sequencer kit (Illumina)	♢ Co-existing *KRAS* and *GNAS* mutations in LAMN (35%) and LAMN + PMP (83%) ♢ High rates of *GNAS* and *KRAS* mutations in LAMN, higher in LAMN + PMP ♢ Wnt/β-catenin pathway activation is a possible mechanism for progression of LAMN to HAMN
LaFramboise et al. 2019 [65]	*N*=10 (5/10 LAMN, 4/10 moderately differentiated MAC, 1/10 poorly differentiated MAC)	56.6 (range 36–68)	N/A	FFPE tumour and metastatic tissue Manual micro-dissection	Targeted amplicon sequencing using Ion Torrent Personal Genome Machine during emulsion PCR	♢ Activating *KRAS* mutations, important component of RAS pathway ♢ HG tumours have higher mutational load ♢ (*MYC*, *DAXX*, *PIM1*, *POU5F1*, *TP53*, and *MYB*)
Liu et al. 2019 [62]	*N*=1 MAC	63	*F*=1 (100)	FFPE samples Primary site	WES using the TruSeq Rapid Capture Exome Kit on the Illumina NextSeq500 platform	♢ Gene mutations in the fibroblast growth factor receptor family ♢ VEGF mutation- key mediator of angiogenesis in cancer ♢ Activating *KRAS* mutation
Tokunaga et al. 2019 [38]	*N*=183 (66/183 PMP, 44/183 MAC)	PMP=56* (range 30–83) MAC=61* (range 25–82)	PMP: *M*=29 (44) *F*=37 (56) MAC: *M*=20 (45) *F*=24 (55)	FFPE samples of tumour and metastatic tissue	NGS using the NextSeq platform	♢ Higher rates of *KRAS* and *GNAS* mutations in PMP compared to MAC ♢ PMP and MAC microsatellite stable ♢ Low tumour mutational burden
Zhu et al*.* 2019 [39]	*N*=68 (21/68 LAMN, 21/68 moderately differentiated MAC, 26/68 poorly differentiated MAC with signet ring cells)	LAMN= 49.7 ± 12.0 moderately differentiated MAC= 56.7 ± 11.0 poorly differentiated MAC= 57.7 ± 12.3	LAMN: *M*=8 (38) *F*=13 (62) Moderately differentiated MAC: M=12 (57) F=9 (43) Poorly differentiated MAC: *M*=8 (35) *F*=15 (65)	FFPE samples	NGS using the AmpliSeq Cancer Hotspot Panel v2	♢ Co-existing *KRAS* and *GNAS* mutations: 57% in LAMN, 57% in moderately differentiated MAC, 23% in poorly differentiated MAC ♢ LAMN mainly *KRAS* and *GNAS* mutations ♢ MAC more complex mutation profiles with more gene mutations ♢ Higher rates of *TP53* mutations in MAC
Ang et al*.* 2018 [64]	*N*=374 (320 MAC, 54 PMP)	MAC= 54* PMP= 54*	*M*= 163 (44) *F*= 211 (56)	N/A	Hybrid capture-based sequencing	♢ *KRAS*, *GNAS*, *SMAD4, APC, ARID1A*, and *TP53* gene mutations seen in both PMP and MAC ♢ *ERBB2* seen in PMP only, involved with *KRAS* in activation of RAS/Raf/MAPK pathway
Gleeson et al. 2018 [31]	*N*=54 PMP (29/54 DPAM, 16/54 PMCA-I, 9/54 PMCA)	Range 47–65	*M*= 22 (41) *F*= 32 (59)	FFPE samples	NGS using Illumina MiSeq platform or Illumina NextSeq platform Sanger sequencing using BigDye Terminator v1.1	♢ Co-existing *KRAS* and *GNAS* mutations (87%), but they do not determine tumour grade ♢ DPAM and PMCA-I more complex mutation profiles with more gene mutations ♢ *TP53* and *NRAS* mutations only in PMCA
Pengelly et al. 2018 [63]	*N*=5 LAMN	Range 35–65	*M*=1 (20) *F*=4 (80)	FFPE samples Laser-capture micro-dissection	WES on a HiSeq 2500 (Illumina, San Diego, CA)	♢ Co-existing *KRAS* and *GNAS* mutations in whole sample ♢ First report of *RNF43* (stopgain) in PMP; its decreased function leads to increased sensitivity of the cell to Wnt signalling
Wen et al. 2018 [40]	*N*=1 MAC	74	*F*=1 (100)	FFPE samples Punch biopsy or macro-dissection	Capture-based NGS on a HiSeq 2500 (Illumina, San Diego, CA)	Co-existing *KRAS* and *GNAS* mutations
Ang et al. 2017 [70]	*N=*1 PMP from disseminated, well-differentiated MAC	53	*M*=1 (100)	Archived omental tissue	Comprehensive genome profiling	Activating *GNAS* mutation may lead to aberrant activation of cAMP-PKA pathway
Matson et al. 2017 [41]	*N*=1 LAMN	49	*F*=1 (100)	DNA from appendiceal tumour	NGS	Co-existing *KRAS* and *GNAS* mutations
Saarinen et al. 2017 [59]	*N*=9 PMP (6/9 LAMN, 3/9 HAMN)	N/A	N/A	FFPE samples Microdissection Macro-dissection to increase yield of tumour DNA	Exome sequencing using Illumina HiSeq 2000 (San Diego CA) Validation by ultra-deep amplicon sequencing with PCR and MiSeq v2 Illumina	♢ 7 genes implicated in the PKA pathway (including *GNAS*), involved in the overproduction of mucin ♢ 6 genes implicated in the TGF-β pathway, key regulator of cell proliferation
Borazanci et al. 2016 [42]	*N*=352 (317/352 MAC, 28/352 PMP, 7/352 mucinous cystadenocarcinoma)	MAC= 55* (range 20–88) PMP=57* (range 31–77) mucinous cystadeno-carcinoma= 57* (range 40–67)	MAC: *M*=126 (40) *F*=191 (60) PMP: *M*=12 (43), *F*=16 (57) Mucinous cystadeno-carcinoma: *M*=3 (43) *F*=4 (57)	FFPE samples	Sanger sequencing using BigDye Terminator v1.1 and analysed using 3730 DNA Analyzer NGS using Illumina MiSeq	♢ Activation of PI3KA pathway in PMP and MAC ♢ Highest rates of *KRAS* and *GNAS* mutations seen in PMP compared to other subtypes ♢ *KRAS* mutations in all mucinous cystadenocarcinomas
Pietrantonio et al. 2016 [44]	*N*=40 PMP discovery (5/40 HG, 35/40 LG) Validation= 25	Discovery= 52* (range 32–71) Validation=54* (range 30–70)	Discovery: *M*=19 (48) *F*=21 (52) Validation: *M*=8 (32) *F*=17 (68)	N/A	NGS using the Ion Torrent Personal Genome Machine platform	♢ *GNAS* mutations associated with *KRAS* mutations (*P*=0.002) ♢ Co-existing *KRAS* and *GNAS* mutations in 52% of sample ♢ Discovered new mutations in angiogenic tyrosine kinase receptors: *FGFR3* and *PDGFRA* ♢ First documentation of presence of an *LKB1* mutation, activation of mTOR pathway
Pietrantonio et al*.* 2016 [43]	*N*=15 PMP (10/15 LAMN, 5/15 HAMN)	Range 42–68	*M*=9 (60) *F*=6 (40)	FFPE samples from peritoneal deposits Manual microdissection	NGS using the Ion Torrent Personal Genome Machine platform	♢ Co-existing *KRAS* and *GNAS* mutations in 64% of sample ♢ *FGFR3* and *LBK1* occur in the same case with long-lasting benefit
Levine et al. 2016 [68]	*N*=63 appendiceal tumour samples (discovery in 24/63, validation in 39/63)	Discovery=54 Validation=51	Discovery: *M*=17 (71) *F*=7 (29) Validation: *M*=19 (49) *F*=20 (51)	Frozen section	Whole-genome expression microarray (RNA) on the Affymetrix U133A GeneChip system	Identification of 139 prognostic gene signature in LG tumours, dividing both study cohorts into two molecular subtypes (measured by disease-specific survival and prognosis-free survival): favourable prognostic molecular subtype and poor prognostic molecular subtype
Wu et al*.* 2015 [58]	*N*=18 (6/18 mucinous appendiceal cystadeno-carcinoma and 12/18 mucinous appendiceal cystadenoma)	Mucinous cystade-noma=55* (range 38–94) mucinous cystadeno-carcinoma=65* (range 35–85)	Mucinous cystadenoma *M*=4 (33) *F*=8 (66) Mucinous cystadeno-carcinoma: *M*=1 (17) *F*=5 (83)	FFPE samples Macrodissection	Comprehensive miRNA microarray expression profiling using miRbase version 21 Validation by quantitative reverse transcriptase PCR using TaqMan miRNA reverse transcription kit and TaqMan universal PCR Master Mixture Kit	♢ Expression of miR-1, miR-4328, miR-200b, miR-200C, miR-223, miR-21, and miR-451 significantly dysregulated in appendiceal mucinous cystadenocarcinoma compared to mucinous cystadenoma ♢ Can be used as molecular biomarkers for tumour diagnosis and provide new therapeutic targets for treatment of cystadenocarcinoma
Noguchi et al. 2015 [26]	*N*=18 PMP (10/18 DPAM, 8/18 PMCA)	64.6 ± 12.8 (range 31–82)	*M*=9 (50) *F*=9 (50)	Frozen sections Laser-capture micro-dissection Primary tumour (12), metastatic lesion (6)	Amplicon sequencing with multiplex PCR using Ion AmpliSeq Cancer Panel v2	♢ Frequent *KRAS* and *GNAS* mutations ♢ *TP53*, *PIK3CA*, and *AKT1* mutations seen in PMCA only ♢ Mutations in *TP53* and genes related to the PI3K-AKT pathway may lead to malignant features of PMP
Hara et al. 2015 [47]	*N*=16 (7/16 LAMN 4/16 LAMN + PMP, 4/16 MAC, 1/16 MAC + PMP)	58 ± 14.60 (range 36–86)	*M*=2 (13) *F*=14 (88)	FFPE tumour samples LAMN: manual microdissection MAC: laser-capture microdissection	PCR followed by direct sequencing	♢ LAMN and MAC both have mutations in *KRAS*, *GNAS*, and *TP53* ♢ MACs can originate from LAMNs as well as *de novo* occurrence ♢ *CTNNB1* mutation in LAMNs-activated Wnt pathway
Roberts et al. 2015 [71]	*N*=4 PMP	N/A	N/A	Fresh frozen samples (3 disseminated [omentum], 1 from primary appendiceal tumour) Laser capture microdissection	Exon array analysis using Affymetrix Genechip Human Exon 1.0 ST Arrays	♢ *SLC16A4* most significantly overexpressed, appears to be PMP-specific ♢ *MS4A12* downregulated despite being a known *CDX2* transcriptional target (CDX2 upregulated in PMP); may reflect different pathways initiated by *CDX2* in appendiceal tissue ♢ ALDOB overexpression may reflect the high metabolic requirements to sustain the production of large amounts of mucin
Nummela et al. 2015 [45]	*N*=19 PMP (9/19 LG, 10/19 HG)	N/A	N/A	FFPE samples Micro-dissection Macro-dissection to increase yield of tumour DNA	NGS with the TruSeq Amplicon Cancer Panel assay using the MiSeq system with the V2 MiSeq SBS kits	♢ High expression of *KRAS* and *GNAS* mutations ♢ Mutations only in HG: *SMAD4*, *TP53*, *AKT1*, and *ATM* ♢ *TP53* expression associated with HG and poorer survival
Alakus et al. 2014 [4]	*N*=29 (discovery in 1/29 HG MCP, 9/29 LG MCP; validation in 5/29 HG MCP, 11/29 LG MCP, 3/29 LAMN)	N/A	N/A	Fresh frozen (discovery) FFPE (validation)	Whole-exome sequencing by HiSeq 2000 Ultra deep targeted sequencing by HiSeq 2000 Confirmation of *KRAS* and *GNAS* mutations by digital droplet PCR assays	♢ Co-existing *KRAS* and *GNAS* mutations in 69% of sample ♢ HG are *GNAS* wild type, indicating they do not progress from LG ♢ HG compromised the highest mutation rate ♢ PKA activation in HG
Davison et al*.* 2014 [48]	*N*=109 (42/109 LAMN, 47/109 moderately differentiated MAC, 20/109 poorly differentiated MAC with signet ring cells)	Range 24–84	*M*=51 (47) *F*=58 (53)	Tumour tissue Manual micro-dissection	PCR	LOH in *SMAD4* in low frequencies across all histology groups, implicated in the TGFβ pathway
Liu et al. 2014 [46]	*N*=24 (15/24 LAMN, 8/24 low-grade/well-differentiated MAC with PMP, 1/24 MAC)	N/A	N/A	FFPE samples Surgical resections and cell blocks of peritoneal fluid Macro-dissection	NGS on the Ion Torrent Personal Genome Machine (PGM^TM^) using Ion 318^TM^ chips	Co-existing *KRAS* and *GNAS* mutations in 40% of LAMN *TP53* only in MAC
Singhi et al. 2014 [49]	*N*=55 (23/55 LAMN, 19/55 moderately differentiated MAC, 13/55 poorly differentiated MAC with signet ring cells)	N/A	*M*=35 (64) *F*=20 (36)	FFPE tumour tissue Manual micro-dissection	PCR using BigDye Terminator v3.1 cycle sequencing kit	♢ *GNAS* frequently mutated in LAMN and moderately differentiated MAC but not in MACs with a predominance of signet ring cells ♢ *GNAS*-mutated neoplasms more frequently harboured concurrent *KRAS* mutations compared with *GNAS* wild-type tumours
Shetty et al. 2013 [69]	*N*=64 PMP (25/64 LG, 39/64 HG)	51* ± 12	*M*=33 (52) *F*=31 (48)	Unstained paraffin sections of tumour tissue Manual micro-dissection	Shifted termination assay (commercial assay) using Muctector 2	♢ High rate of *KRAS* mutations located on codon 12 ♢ *KRAS* mutations do not influence prognosis
Pulighe et al. 2013 [72]	*N*=1 appendiceal mucinous cystadenoma	68	*M*=1 (100)	N/A	Direct sequencing using Applied Biosystems	*PIK3CA* mutation, activation of PI3K pathway
Nishikawa et al*.* 2013 [61]	*N*=35 (32/35 LAMN, 3/35 MAC)	N/A	N/A	FFPE tumour samples for all MACs and 28 LAMNs, FFPE peritoneal or omental deposits 4 LAMNs Macro-dissection	Sanger sequencing using Applied Biosystems 3130 Genetic Analyser	♢ Frequent activating *GNAS* mutations in LAMN but not in MAC ♢ Activation of cAMP-PKA pathway
Zauber et al. 2011 [50]	*N*=31 LAMN	56.7 (range 33–85)	*M*=8 (26) *F*=23 (74)	FFPE samples	Sanger sequencing for KRAS using BigDye Terminator cycle sequencing kit PCR for microsatellite analysis and LOH of APC gene using ABI 9700 thermal cycler	♢ *KRAS* mutations in whole sample of LAMN ♢ All LAMNs are microsatellite stable ♢ LAMNs did not have LOH of the *APC* gene
Maheshwari et al. 2006 [51]	*N*=23 (6/23 DPAM, 7/23 PMCA-I, 10/23 PMCA)	53.6* (range 27–90)	*M*=17 (74) *F*=6 (26)	Microdissected tissue samples	PCR	♢ Fractional mutation rate (FMR) <.25 predictive of DPAM ♢ FMR between .25 and .50 correlated with PMCA-I ♢ FMR >.50 predictive of PMCA ♢ Increasing FMR correlated with poorer prognosis
Sebastian et al. 2006 [73]	*N*=1 MAC	75	*F*=1 (100)	Frozen section	Analysis of clonal nature	*KRAS* mutation, activation of the RAS/Raf/MAPK pathway
Feltmate et al. 2005 [57]	*N*=14 MAC	Range 36–84	N/A	Frozen samples	Whole-genome amplification using Genomiphi DNA amplification system LOH analysis by PCR	MAC had significantly higher LOH rates at locus D6S462 on chromosome 6q21 (*P*=0.0183) and locus DXS1226 on chromosome Xp11.4 (*P*=0.0366) than mucinous ovarian carcinomas
Maru et al. 2004 [52]	*N*=17 MAC	58.3 ± 11.25	*M*=10 (59) *F*=7 (41)	FFPE tumour samples Microdissection	PCR using BigDye Terminator Cycle Sequencing Ready Reaction Kit	Frequent chromosome 18q loss Low rate of *SMAD4* mutation (located on chromosome 18q), increased signal transduction in the TGFβ pathway
O’Connell et al. 2002 [74]	*N*=100 PMP (70/100 DPAM, 20/100 PMCA-I, 10/100 PMCA) *N*=15 solitary mucinous tumours of appendix without PMP	N/A	N/A	N/A	*In situ* hybridisation	♢ *MUC2* uniformly expressed in PMP ♢ High expression of *MUC5AC* in PMP ♢ *MUC2* and *MUC5AC* expressed in all solitary mucinous tumours ♢ Potential role for *MUC2* as a reliable molecular marker in PMP
Kabbani et al. 2002 [53]	*N*=23 (22/23 LAMN, 1/23 MAC) (16 with PMP)	56 ± 13	*M*=17 (75) *F*=6 (25)	FFPE tumour samples Micro-dissection	PCR followed by sequencing using BigDye Terminator Cycle Sequencing Ready Reaction Kit	♢ *KRAS* mutation in half of samples- RAS/Raf/MAPK pathway ♢ All samples were microsatellite stable
Shih et al. 2001 [54]	*N*=2 (1 DPAM, 1 mucinous adenoma with no PMP)	32	*M*=2 (100)	FFPE samples of appendiceal and peritoneal tumours Micro-dissection under inverted microscope	Digital PCR to analyse KRAS mutations using Applied Biosystems Big Dye terminators and Applied Biosystems 377 automated sequencer Digital single nucleotide polymorphic assay to analyse LOH of APC	♢ Different variations of *KRAS* mutations seen in both DPAM and mucinous adenoma ♢ LOH of *APC* seen only in mucinous adenoma
Szych et al. 1999 [55]	*N*=17 PMP Additional *N*=16 mucinous adenoma	N/A	N/A	FFPE samples Micro-dissection under a light microscope	PCR using the ThermoSequenase radiolabeled terminator cycle sequencing kit	♢ LOH at chromosome 5q most frequently detected in PMP ♢ LOH of 18q, 5q, 17p, and 6q frequently detected in appendiceal lesions in PMP compared to ovarian lesions ♢ Suggests that PMP is of appendiceal origin
Chuaqui et al. 1996 [56]	*N*=12 appendiceal mucinous lesions (8/12 without invasion, all with PMP, 2/12 invasive, one with PMP one without)	56.5 (range 40–78)	N/A	FFPE samples Micro-dissection under direct light microscope visualisation	PCR	LOH at 5q in appendiceal lesion and not ovarian, supports diagnosis of two independent primary mucinous lesions

*Indicates median age; *DPAM*, disseminated peritoneal adenomucinosis; *FFPE*, formalin-fixed paraffin embedded; *LAMN*, low-grade appendiceal mucinous neoplasms; *LG*, low grade; *LOH*, loss of heterozygosity; *HAMN*, high-grade appendiceal mucinous neoplasms; *HG*, high grade; *MAC*, mucinous adenocarcinoma; *N/A*, data not available; *NGS*, next-generation sequencing; *PCR*, polymerase chain reaction; *PMCA-I*, peritoneal mucinous carcinomatosis-intermediate; *PMCA*, peritoneal mucinous carcinomatosis; *PMP*, pseudomyxoma peritonei; *SD*, standard deviation

### Prevalence of mutations

The most frequently identified somatic gene mutations were in *KRAS* and *GNAS* with 70% of studies (*n*=32) identifying *KRAS* gene mutations (a proto-oncogene) and 57% of studies (*n*=26) identifying *GNAS* gene mutations (a complex locus) (Table 2). Across the reviewed studies, the most common variants noted in *KRAS* mutations were found on codon 12 (G12D, G12C, and G12V) and in codon 13 (G13D) in PMP and AMN [30, 44, 74]. The most commonly identified *GNAS* mutation variants were R201H and R201C [30]. Additional oncogenes were identified: 24% of studies (*n*=11) identified *PIK3CA* mutations, 11% (*n*=5) identified *CTNNB1* mutations, and 13% (*n*=6) identified *AKT1* mutations. The following tumour suppressor genes were identified: 37% of studies (*n*=17) identified *TP53* mutations, 30% (*n*=14) identified *SMAD4* mutations, 24% (*n*=11) identified *APC* mutations, 9% (*n*=4) identified *ATM* mutations, 9% identified *RNF43* mutations, and 7% (*n*=3) identified *RB1* mutations. A summary of all genetic alterations identified are recorded in the Supplementary Table 2.

All papers stated the experimental method used to analyse their samples (Table 2), and the two most frequently used methods were next-generation sequencing (NGS) and polymerase chain reaction (PCR) across studies. A third of studies (*n*=15) used NGS as their main experimental method [32–35, 38, 41, 42, 44, 46, 48, 50–52, 57, 59]. A third of studies (*n*=15) used PCR: 13 studies used it as their main experimental method [4, 26, 47 57] and 2 studies used it to validate their findings [49, 54]. Some studies (13%; *n*=6) used Sanger sequencing [34, 36, 44, 50, 63, 64] and 7% of studies (*n*=3) used whole-exome sequencing [4, 40, 45]. All methods have been recorded in Table 2.

A third of studies (*n*=14) noted co-existing *KRAS* and *GNAS* mutations in their samples with co-occurrence rates ranging from 13 to 100% [4, 30, 32, 34, 35, 38, 42, 44–46, 48, 51, 52, 59] (Table 2). Some studies discovered co-occurrences of *KRAS* and *GNAS* mutations in both low-grade and high-grade lesions of PMP and AMN by NGS in formalin-fixed paraffin-embedded (FFPE) samples [4, 30, 32, 34, 44, 51, 52]. For example, Yanai et al. [34] used NGS on FFPE samples to identify a co-occurrence rate of 15% in LAMN, 13% in PMP cases caused by HAMN, and 22% in MAC. Pietrantonio et al*.* [51] supports this as they found that *GNAS* mutations were significantly associated with *KRAS* mutations (*P*=0.002) and noted a co-occurrence rate of 52% in their PMP samples. Flatmark et al. [30] used NGS on FFPE samples and noted co-occurrences in 87% of their low-grade PMP and 100% of their high-grade PMP samples. Two studies did not specify the rates according to histological subtype in their samples of PMP; however, Gleeson et al*.* [44] found a co-occurrence rate of 87% and Pietrantonio et al*.* [51] found a co-occurrence rate of 64%.

Other studies comprised of only low-grade samples also documented the co-occurrence of *KRAS* and *GNAS* mutations using the same experimental methods [35, 38, 42, 45, 48, 59]. Using NGS, Foster et al*. *[35] noted a co-occurrence rate of 100% in their sample of LGMCP, Tsai et al*. *[38] identified a co-occurrence rate of 35% in LAMN and 83% in PMP cases caused by LAMN, and Liu et al*.* [59] identified a co-occurrence rate of 40% in their sample of LAMN. Pengelly et al*.* [45] identified a co-occurrence rate of 100% in their sample of LAMN using whole-exome sequencing, and Alakus et al*.* [4] noted a co-occurrence rate of 69% in their sample of LAMN and PMP using PCR. There were two case reports that used NGS: Matson et al*.* [48] found that both *KRAS* and *GNAS* were present in their patient with LAMN (*n*=1), and Wen et al*.* [46] found the same in their patient with MAC (*n*=1).

The low co-occurrence rate in MAC has also been established by Liao et al. [32] who noted the co-occurrence to be 63% in LAMN, 56% in HAMN, and 10% in MAC. Zhu et al*.* [42] supports this as they noted the co-occurrence to be 57% in LAMN, 57% in moderately differentiated MAC, and 23% in poorly differentiated MAC. The co-occurrences of these mutations in multiple studies indicate that they are a common characteristic of PMP. This suggests that whilst there may be a relationship between LAMN and HAMN on a molecular level, there are other gene mutations that are responsible for the progression from LAMN to HAMN such as *TP53*, and this distinguishes these neoplasms from MAC which have markedly lower expression of *KRAS* and *GNAS* mutations.

Studies (*n*=17) noted the presence of *TP53* mutations in PMP and AMN samples [4, 26, 32, 34, 35, 38–44, 51, 52, 55, 57, 59]. Nine papers noted *TP53* expression in only high-grade neoplasms and PMP [4, 26, 32, 38–40, 44, 57, 59], whereas four papers noted *TP53* expression in both low-grade and high-grade neoplasms [34, 35, 42, 55] (Table 2). Specifically, Nummela et al. [57] noted that P53 protein expression was significantly associated with HAMN (*P*=0.012). In studies where *TP53* was expressed exclusively in PMP caused by high-grade neoplasms, this indicated that this mutation led to more malignant phenotypes [26, 44]. This was expected as high-grade neoplasms harboured more gene mutations [42, 44] and exhibited more malignant cytology compared to low-grade neoplasms [2]. Some studies did not stratify their findings into histological subtype, and therefore it was not possible to report whether *TP53* mutations were exclusive to a specific tumour grade [41, 43, 51, 52].

### Prognostic value of mutations and molecular subtypes

Two studies identified molecular subtypes according to gene expression patterns and stratified patient samples into immune-enriched, oncogene-enriched, or mixed subtypes [31, 37] (Table 2). The oncogene-enriched subtype was characterised by overexpression of genes associated with cancer progression including *ERBB2*, *SLC44A4*, and *EPCAM*, whereas the immune-enriched subtype was marked by overexpression of genes with roles in immune pathways including *IL23A*, *TRA*, and *CD37*. The mixed subtype was characterised by overexpression of genes from both categories. The oncogene-enriched subtype had a significantly lower OS of 1.4 years than the immune-enriched (7.7 years) and mixed subtypes (3.6 years) (*P*=0.005), and the molecular subtypes were independent predictors for survival [31, 37]. This was expected as oncogene-enriched subtype tumours exhibited more aggressive tumour growth and had an increased expression of gene mutations involved in cell proliferation (*ERBB2*, *SLC44A4*, *EPCAM*, *CLDN3*, and *CLDN4*), cell differentiation (*ELF3* and *GPX2*), and mucin production (*KRT20*).

Levine et al*.* [53] developed a 2-tier prognostic molecular subtype classification based on the gene expression profile of 139 genes in LGMCP. Unsupervised hierarchical clustering analysis identified two molecular subtypes based on gene expression patterns and their association with survival outcomes. These were denoted ‘favourable-prognosis subtype’ marked by increased expression of pathway signatures reflecting allograft rejection and antigen processing and presentation, and ‘poor-prognosis subtype’ marked by overexpression of proto-oncogenes including *EPCAM*, *CEACAM5*, *FGFR3*, *HER-2*, and *MET*. They noted that a subset of LGMCP predominantly associated with the poor-prognosis subtype group did not respond to CRS/HIPEC. The higher failure rate indicated there was a potential role for adjuvant, systemic therapies in this subset of patients which may be determined according to the gene mutation profiles.

Out of the 46 studies, 16 studies (35%) undertook survival analyses (Table 3). Pietrantonio et al*.* [51] noted that *KRAS* mutation status was an independent predictor of PFS in a multivariate analysis. However, other studies found no significant difference in the OS in *KRAS*-mutated neoplasms compared to *KRAS* wild-type, and *KRAS* mutations were not an independent prognostic factor for OS [60, 61]. Although two studies noted that samples with *GNAS* mutations had significantly shorter median PFS [51, 52], one study found no significant difference in OS in tumours with *GNAS* mutations compared to those without [60]. Additionally, samples with loss of *SMAD4* expression had a significantly lower OS than those with preserved expression but it was not an independent factor of the same [58]. Three studies agreed that *TP53* mutations and p53 expression were associated with poorer PFS and OS in log-rank tests [42, 57, 61]. However, they did not reach a consensus on their effect as independent predictors in multivariate analyses [42, 57]. One study noted that patients with relapsed PMP receiving palliative treatment of combined metronomic capecitabine and bevacizumab achieved a median PFS of 8.2 months with a 1-year OS of 91% [52]. Whilst this suggests that the drugs were effective, it would be beneficial to compare the effect of this combination treatment with a cohort that did not receive the drugs or with other drug treatments to make accurate comparisons on the true efficacy of the treatment and compare survival endpoints. Lastly, five studies concluded that tumour grade was an independent prognostic factor for survival [37, 42, 53, 58, 60]. However, Nummela et al. [57] did not find this to be statistically significant in their multivariate analysis. Tumours should be graded with the same classification system, and survival analyses should be undertaken for each histological subtype separately to allow for accurate comparisons on the role of individual gene mutations on survival to be made. The survival analysis findings have been summarised in Table 3.

**Table 3:** Survival analysis for the studies included in this systematic review

Study	Sample	Type of survival analysed	Median follow-up period (months)	Analysis	Results	Implication
Moaven et al*.* 2020 [31]	*N*=79 (17/79 Immune enriched [13 LG, 4HG] 35/79 mixed [21 LG, 14HG], 27/79 oncogene enriched [14 LG, 13HG])	OS	N/A	Kaplan-Meier and log-rank survival curves Cox proportional hazard models in LG and HG for univariate and multivariate analysis *P*<0.05	Median survival for whole cohort (years):Immune-enriched=7.7, mixed=3.6, oncogene-enriched=1.4 (*P*=0.005) Immune-enriched vs mixed (median 3.6 years, HR 2.08, 95% CI 0.94–4.55) Immune-enriched vs oncogene-enriched (median 1.4 years, HR 3.64, 95% CI 1.63–8.13) *P*=0.005 Oncogene-enriched vs immune-enriched HR 3.64, 95% CI 1.63–8.13, *P*=0.0017 Low-grade: median (years): immune-enriched=7.7, mixed=6.1, oncogene-enriched=5.1 Immune-enriched vs oncogene-enriched HR 3.55, 95% CI 1.10–11.4, *P*=0.033 High-grade: Median (years): immune-enriched=2.3, mixed=1.6, oncogene-enriched=1.6 Immune-enriched vs oncogene-enriched HR 3.59, 95% CI 1.08–11.9, *P*=0.037	♢ Immune-enriched subtype had the highest median survival followed by mixed and oncogene-enriched subtypes which was significant ♢ Oncogene-enriched subtype survival rate is significantly lower than immune-enriched subtype ♢ Immune-enriched had a significantly better OS than oncogene-enriched in low-grade and high-grade neoplasms
Yanai et al. 2020 [34]	*N*=51 (28/51 LAMN, 6/51 LAMN+PMP1/51 HAMN, 7/51 HAMN +PMP, 8/51 MAC, 1/51 MAC+PMP)	OS RFS	N/A	Kaplan-Meier and log-rank	OS MAC vs combined LAMN and HAMN *P*=0.034 RFS MAC vs LAMN *P*=0.017 HAMN vs LAMN *P*=0.017	♢ MAC showed a significant shorter OS rate than LAMN/HAMN ♢ MAC had significantly shorter RFS than LAMN ♢ HAMN had significantly shorter RFS than LAMN
Foster et al. 2020 [35]	*N*= 11 LGMCP	OS	112	Kaplan-Meier and log-rank Cox proportional hazard model	Median OS: 111.8 months LGMCP vs high-grade neoplasms, *P*<0.0001	♢ LGMCP had a significantly better median OS compared to high-grade neoplasms
Su et al. 2020 [37]	*N*=138 (76/138 LG, 38/138 HG)	OS PS	N/A	Kaplan-Meier and log-rank tests for non-parametric survival analysis Cox proportional hazard models for univariate and multivariate analysis	OS (subtypes): log-rank *P* <0.0001 PFS (subtypes): log-rank *P* 0.0002 Oncogene-enriched subtype: 3 year OS: 36% (95% CI 24–55%), median 1.4 3 year PFS: 14% (95% CI 5.0–41%) median 0.9 Multivariate analysis for OS: HR 2.5, 95% CI 1.0–6.2, *P*=0.044 Mixed subtype: 3 year OS: 55% (95% CI43–72%), median 3.4 3 year PFS: 30% (17–53%) median 1.5 Immune-enriched subtype: 3 year OS: 83% (95% CI 71.5–96.5%) 3 year PFS: 65.5% (95% CI 51–84%), median 4.1 Grade Multivariate analysis for OS: HR 6.6, 95% CI 2.8–15, *P*<0.0001	♢ OS and PFS were significantly different for the molecular subtypes ♢ Oncogene-enriched subtype was an independent predictor of OS, tumours with oncogene-enriched subtype have poorer OS ♢ Grade was an independent predictor of OS ♢ Immune-enriched subtype highest median PFS, followed by mixed subtype and oncogene-enriched subtype had the lowest median PFS
LaFramboise et al. 2019 [39]	*N*=10 (5 G1, 4 G2, 1 G3)	OS PFS	N/A	N/A	Median OS (months) G1: 54.8 ± 6.2 G2: 43.8 ± 25.6 G3: 22.7 Median PFS (months) G1: 41.5 ± 9.1 G2: 30.5 ± 21.7 G3: 17.7 High-grade OS=36.8±12.7 months Low-grade OS=53±4.7 months	♢ Low-grade tumours had the highest median OS ♢ 4/5 patients with high-grade neoplasms suffered recurrence ♢ 4/5 patients with low-grade neoplasms recurrence free at 4-year follow-up
Zhu et al*.* 2019 [42]	*N*=56 (14/21 G1, 21/21 G2, 21/26 G3)	OS PFS	40 months	Kaplan-Meier method and Cox proportional hazards model *P*<0.05	Multivariate analysis: G1 vs G2 *P*=0.0369 G1 vs G3 *P*=0.0273 G1 compared with combined G2/G3 OS: HR 2.32, 95% CI 1.07–5.03, *P*=0.0329 PFS: HR 3.3, 95% CI 1.42–7.63, *P*=0.0054 Mutation: *TP53* PFS in G2 tumours *P*=0.0268 PFS in Combined G2/G3: *P*=0.0315	♢ G1 tumours significantly better survival compared to G2 and G3 tumours ♢ Grade was an independent prognostic factor for OS + PFS ♢ *TP53* significant adverse risk factor for PFS in G2 tumours ♢ Poorer PFS in TP53-mutated tumours compared to wild-type *TP53* tumours for combined G2/G3
Ang et al. 2018 [43]	*N*=76 (PMP=13, MAC=33)	OS	29.9	Kaplan-Meier plots and log-rank	PMP vs signet ring cell carcinoma: *P*=0.11 MAC: *P*=0.29	♢ PMP had a better OS compared to signet ring cell carcinoma although not significant
Pietrantonio et al. 2016 [51]	*N*=25	PFS	68.1	Kaplan-Meier and log-rank tests Cox proportional hazard model for univariate and multivariate analysis	*GNAS* mutation: Univariate analysis: HR 3.06, 95% CI 1.29–7.27 *P*=0.011 *KRAS* mutation: Univariate analysis: HR 17, 95% CI 2.33–134.40 *P*=0.006 Multivariate analysis: HR 15.09, 95% CI 1.80–126.27 *P*=0.012 5-year PFS= 41.2% months Median PFS= 37.4% months 5-year OS= 89% months Median OS not reached	♢ *KRAS* was an independent predictor of PFS ♢ Decreased PFS in *KRAS*-mutated tumours ♢ Shorter median PFS in *GNAS*-mutated tumours
Pietrantonio et al. 2016 [52]	*N*=15 PMP (10/15 LAMN, 5/15 HAMN)	PFS OS	PFS: 12 (range 3–18)	Kaplan-Meier method and log-rank test	1 year OS= 91% (95% CI 75%-100%), median OS not reached Median PFS: *GNAS* mutation *GNAS* mutations (5.3 months) vs *GNAS* wild-type (not reached) HR 7.57, 95% CI 1.73–33.20, *P*=0.007	♢ Median PFS significantly shorter in patients with *GNAS* mutations in comparison with *GNAS* wild-type status
Levine et al. 2016 [53]	*N*=63 (discovery in 24/63, independent cohort 39/63)	DSS PFS	N/A	Kaplan-Meier and log-rank Cox proportional hazards regression for univariate and multivariate analysis	2 molecular subtypes of prognostic value (favourable vs poor) DSS *P*=0.0075 PFS *P*=0.0072 Subtypes (DSS): Univariate: HR 3.64, 95% CI 1.53–8.70, *P*=0.004 Multivariate: HR 3.53 95% CI 1.41–8.85, *P*=0.007 Grade (DSS): Univariate HR 6.24, 95% CI 2.56–15.23, *P*<0.001 Multivariate HR 6.84, 95% CI 2.57–18.17, *P*<0.001 Subtypes (PFS): Univariate HR 3.16, 95% CI 1.64–6.10, *P*<0.001 Multivariate HR 3.27, 95% CI 1.58–6.75, *P*=0.001 Grade (PFS): Univariate HR 3.52, 95% CI 1.71–7.23, *P*<0.001 Multivariate HR 3.32, 95% CI 1.57–7.01, *P*=0.002 Independent cohort: DSS= 0.034, PFS=0.0079, Low-grade only: DSS= 0.028, PFS=0.0016	♢ Poor-prognosis subtype had a significantly worse DSS and PFS compared to favourable-prognosis subtype ♢ Molecular subtypes were independent prognostic factors in both DSS and PFS ♢ Tumour grade independent prognostic factor in both DSS and PFS ♢ In independent cohort, molecular subtypes stratified patients into significantly different outcome groups, high gene-expressing subtype associating with poor outcomes
Nummela et al. 2015 [57]	*N*=68	OS	N/A	Kaplan-Meier method and log rank Cox proportional hazards model for univariate and multivariate analysis	p53 expression: OS (5-year) aberrant p53 staining (19.0%) vs normal staining (79.7%), *P*=0.001 Univariate: HR 4.512, 95% CI 1.657–12.287 *P*=0.003 Multivariate: HR 11.941, 95% CI 1.274–14.856 *P*=0.002 LG vs HG: 5 year survival in HG (53.1%) vs LG (86.6%), *P*=0.009	♢ OS of patients showing aberrant p53 staining was significantly worse than those having normal staining ♢ p53 is an independent prognostic factor for reduced OS ♢ HG had a significantly lower 5-year survival than LG
Davison et al*.* 2014 [58]	*N*=103 (40/42 G1 AKA LAMN, 44/47 G2 AKA moderately differentiated MAC, 19/20 G3 AKA poorly differentiated MAC)	OS	N/A	Kaplan-Meier and log rank test Cox proportional hazard regression for multivariate analysis	Loss of *SMAD4* expression: Loss of expression vs preserved expression *P*=0.023 5-year OS loss of *SMAD4* expression= 49% 5 year OS preserved expression= 68% Univariate analysis: Loss of *SMAD4* expression: HR 2.72, 95% CI 1.11–6.66 *P*=0.029 AJCC tumour grade Univariate analysis: *P*<0.001 G1 vs G2 HR 5.4, 95% CI 2.18–13.38 G1 vs G3 HR 7.11, 95% CI 2.56–19.76 Multivariate analysis: *P*<0.001 G1 vs G2: HR 4.91, 95% CI 1.86–13.0 G1 vs G3: HR 10.3, 95% CI 3.23–32.7	♢ Tumours with loss of *SMAD4* expression had significantly worse OS in comparison with preserved *SMAD4* expression ♢ AJCC grade was an independent predictor to OS, higher grades (G2/G3) had poorer OS compared to G1
Singhi et al. 2014 [60]	*N*=55 (23/55 G1 AKA LAMN, 19/55 G2 AKA moderately differentiated MAC, 13/55 G3 AKA poorly differentiated MAC with signet ring cells)	OS	47	Kaplan-Meier method and log-rank test Cox proportional hazards model for univariate analysis and multivariate analysis	AJCC grade Multivariate analysis: *P*=0.01 G1 vs G2: HR 5.0, 95% CI 1.3–19.0 G1 vs G3: HR 6.1, 95% CI 1.3–28.3	♢ Only tumour grade was an independent predictor of OS, high tumour grade (G2/G3) had poorer OS
Shetty et al. 2013 [61]	*N*=64 PMP (25/64 LG, 39/64 HG)	OS	KRAS: 39 P53: 47	Kaplan-Meier and log-rank test	P53 overexpression Median survival: 89 ±12 months (no overexpression) vs 71 ± 12 months (overexpression), *P*=0.04	♢ P53 overexpression associated with significantly worse OS
Maheshwari et al. 2006 [65]	*N*=23 (6/23 DPAM, 7/23 PMCA-I, 10/23 PMCA)	OS	N/A	N/A	Whole cohort: Median OS 34 months Mean OS 43.9 months	♢ DPAM had a higher OS than PMCA-I and PMCA
Kabbani et al. 2002 [70]	*N=*30 (23 mucinous and 7 nonmucinous carcinomas)	OS disease-free survival	N/A	Kaplan-Meier	Mean OS mucinous carcinoma 26 ± 19 months vs nonmucinous carcinomas 13 ± 9 months (*P*=0.0002) Mean disease-free survival mucinous carcinomas 18 ± 3 months vs nonmucinous carcinomas 7 ± 4 months (*P*=0.04)	♢ Patients with mucinous carcinoma had a significantly better OS and disease-free survival

Bold *P* values are statistically significant; *P*<0.05 indicates statistical significance. *DSS*, disease-specific survival; *DPAM*, disseminated peritoneal adenomucinosis; *G1*, low-grade appendiceal mucinous neoplasm; *G2*, moderately differentiated mucinous adenocarcinoma; *G3*, poorly differentiated mucinous adenocarcinoma with signet ring cells; *N/A*, data not recorded; *OS*, overall survival; *PFS*, progression-free survival; *PMCA*, peritoneal mucinous carcinomatosis; *PMCA-I*, peritoneal mucinous carcinomatosis-intermediate; *RFS*, relapse-free survival

### Risk of bias

Overall, across all four domains, 41% (*n*=19) papers had a low risk of bias, and 59% (*n*=27) had a moderate risk of bias due to conflicts with the confounding domains (Supplementary Table S2). Two studies did not specify the origin of their tumours as their samples consisted of synchronous mucinous tumours of appendiceal and ovarian origin [72, 73]. Other studies (*n*=25) had issues with tumour grading: 12 did not specify what classification system was used [4, 35, 36, 41, 47, 50–52, 54, 59, 62, 66], five used older versions of the World Health Organization (WHO) classification system [49, 57, 67, 68, 70], four used the Bradley classification [31, 37, 53, 61], two used older versions of the American Joint Committee on Cancer Staging Manual [58, 60], one used the Misdraji classification [63], and one used the TNM classification of malignant tumours [40]. Due to the variations in nomenclature, 18 out of these 25 studies had a moderate reporting bias as they stratified their results according to tumour grades, which may have influenced the subsequent analyses. All studies in the systematic review had a low risk of selection and information bias.

## Discussion

This systematic review included 46 studies and found *KRAS*, *GNAS*, and *TP53* were the most frequently identified somatic gene mutations with other genetic alterations recorded in lower frequencies. Moreover, three papers identified molecular subtypes based on gene signatures that had prognostic value [31, 37, 53]. The mean age recorded in these papers ranged from 51 to 68 years, and there was an overall female predominance (≥51%) in their samples. Although the WHO does not have global statistics on PMP or AMN, Orphanet (the portal for rare diseases and orphan drugs) states that PMP has a female predominance with an age of onset after 40 years which shows that these studies represent global data [75]. There is an unmet need to establish rational treatment options for PMP patients for which the current gold standard CRS-HIPEC does not offer a cure of the natural disease progression.

### KRAS, GNAS, and TP53 mutations in PMP and AMN

*KRAS* is a proto-oncogene that codes for the RAS involved in the RAS/Raf/MAP-kinase pathway regulating cell differentiation, proliferation, and apoptosis (Supplementary Figure S1) [76, 77]. RAS has also been noted to activate other pathways such as the PI3K-AKT-mTOR pathway which is involved in promoting cell growth and suppressing apoptosis [78] (Supplementary Figure S1A). Most common mutations in *KRAS* associated with cancer result in aberrant RAS activation, leading to uncontrolled cell growth, contributing to tumourigenesis [76, 79] (Supplementary Figure S1B) [80]. The high prevalence of *KRAS* mutations across histological subtypes, particularly in PMP (64–100%), confirms that it is a hallmark feature in these neoplasms.

Small molecule inhibitors have been developed to specifically target *KRAS* mutations and have been tested in solid tumour models including colorectal, pancreatic, ovarian, and lung cancer. For example, MRTX849 (adagrasib) is an irreversible covalent inhibitor, selectively binding to *KRAS* G12C (G>T) in its GDP-bound state, keeping the mutated protein locked in its inactive form. As a result, the Raf/MAPK pathway is not activated, preventing tumour growth and proliferation [78]. These were ground-breaking findings as they provided a foundation for targeting this mutation in cancer. This compound has shown promising results in a phase I/II clinical trial for colorectal cancer [81], non-small cell lung cancer [82], and pancreatic adenocarcinoma namely KRYSTAL-1 (ClinicalTrials.gov; ID: NCT03785249) [79]. Other clinical trials have identified sotorasib (ClinicalTrials.gov; ID: NCT03600883) [83, 84], JAB-21822 (ClinicalTrials.gov; ID: NCT05009329), and D-1553 (ClinicalTrials.gov; ID: NCT04585035) as targeted treatments for *KRAS* G12C mutations in solid tumours, non-small cell lung cancer, and colorectal cancer with sotorasib already approved as a treatment for small cell lung cancer. Both variants, *KRAS* G12D and G12C, are missense mutations affecting a commonly mutated codon in PMP. We performed functional annotation of the *KRAS* G12D variant using the Ensembl variant effect predictor (VEP) (https://www.ensembl.org/Tools/VEP, date accessed: 13 October 2022). G12D is predicted to be functionally deleterious (SIFT) and likely to be clinically pathogenic (ClinVar) (Supplementary Table 4). Notably, very recent clinical trials are utilising HRS-4642 (ClinicalTrials.gov; ID: NCT05533463) and ASP3082 (ClinicalTrials.gov; ID: NCT05382559) to specifically target *KRAS* G12D mutations in solid tumours. This supports a rationale to also explore targeting *KRAS* G12D in pre-clinical studies in PMP to produce preliminary results supporting a clinical trial.

In PMP, the character-defining redistribution phenomenon occurs when extracellular mucin follows the normal flow of peritoneal fluid, redistributing the mucin and neoplastic cells [3, 5]. Therefore, targeting mucin production would help prevent metastatic spread. Dilly et al*.* [85] investigated the use of dual MEK-PI3K drug therapy against *KRAS*-mutated mucinous appendiceal and colonic cancers as well as a mucin 2-secreting LS174T cell line. Using co-treatment with trametinib, a MEK inhibitor, and pictilisib, a PI3K inhibitor, they noted that there were reduced phosphorylated-ERK and phosphorylated-AKT protein levels which supported the notion that MAPK/PI3K signalling was inhibited. There was a significant decrease in *MUC2* expression suggesting an effective mucinous tumour growth suppression which may be an effective therapy in these patients.

*GNAS* is a complex locus that codes for the stimulatory alpha subunit of the guanine nucleotide-binding protein complex (G-protein) (Gsα) involved in the protein kinase A (PKA) pathway-regulating metabolism, cell growth, and differentiation (Supplementary Figure S2A) [86, 87]. In PMP and AMN, the variants of *GNAS* mutations are most commonly located on codons R201C and R201H [30]. These variants code for a mutated form of Gsα which will lead to aberrant activation of adenylate cyclase resulting in the constant activation of downstream signalling leading to uncontrolled cell proliferation (Supplementary Figure 2B).

In the reviewed studies, *GNAS* mutations were identified in both low-grade and high-grade neoplasms at high frequencies (ranging 31–100%), and the most common variants were R201C and R201H (Table 2). Studies have indicated that *GNAS* mutations may have a role in mucin production, a key morbidity of PMP [26, 63]. Nishikawa et al*.* [63] noted that *GNAS* mutations were significantly associated with the protein expression of MUC5AC (*P*=0.037) in LAMN and MAC. When introduced into the colorectal cell line HT29, *GNAS* mutations led to a significant increase of MUC2 and MUC5AC expression which are well known for their role in mucin production in PMP. This implicates the PKA pathway as a potential therapeutic target for managing mucinous ascites, a hypothesis that requires further investigation.

A recent study using blood and tumour samples from PMP patients (samples used from Clinical Trials.gov; ID: NCT02073500) [30], identified *GNAS* mutations (R201H and R201C) and suggested the resulting Gsα to be a potential neoantigen. *In vitro* stimulation of PBMCs using peptides containing aforementioned point mutations resulted in strong immune responses as measured by proliferation and IFN-γ production. Additionally, CyTOF analysis of tumour samples revealed expression of immune checkpoint (IC) molecules, particularly TIGIT and PD-1 in infiltrating T cells, suggesting a pre-existing immune response, providing the rationale for combination therapy with IC inhibitors and Gsα peptide vaccine in PMP.

Developing successful treatments that target point mutations per se, has been challenging due to the intrinsic characteristics of the proteins they encode, and targeting strategies have been nonspecific and inefficient [88]. Small molecule inhibitors have shown controversial efficacy, due to lack of activity or selectivity, and significant off target effects [88]. This has become apparent with the unsuccessful efforts to target *TP53* mutations in the past decades [89, 90]. Nevertheless, the recent clinical trials focussing efforts on directly targeting *GNAS* and *KRAS* mutations have shown promising advances with considerable clinical efficacy.

### Molecular subtypes

CRS/HIPEC is the current standard of care treatment for PMP, albeit with high failure rates. Sinukumar et al. [91] noted that only 44% achieved complete cytoreduction with no residual tumour, and Chua et al*.* [92] found that 49% of their sample had recurrence of disease in less than 12 months after surgery. Surgeons make the decision to proceed with incomplete CRS/HIPEC based on their own experience and discretion, but there is no rational consensus or tool to guide this decision in the clinical setting. Three studies identified molecular subtypes in samples with failed or incomplete CRS/HIPEC based on gene expression profiles and found favourable-prognosis, poor-prognosis, oncogene-enriched, immune-enriched, and mixed subtypes with prognostic value [31, 37, 53].

Two studies developed molecular subtypes according to gene expression patterns and stratified their patient samples into immune-enriched, oncogene-enriched, or mixed subtypes [31, 37]. The oncogene-enriched subtype had no long-term survivors as they had a mean OS of 1.4 years [31], which implied that incomplete CRS should be avoided in this subtype as it did not yield a prolonged benefit. By contrast, the immune-enriched subtype had a longer survival with a mean OS of 7.7 years [31] which supported the use of CRS/HIPEC even in the event of incomplete cytoreduction. A future hypothesis may explore the efficacy of preoperative chemotherapy treatment in patients with oncogene-enriched subtypes and preoperative immunotherapy treatment in those with immune-enriched subtypes as adjuvant treatments to CRS/HIPEC to further improve the OS.

Levine et al. [53] developed two different molecular subtypes in LGMCP known as poor-prognosis and favourable-prognosis subtypes. The differences in the molecular subtypes implied that there was heterogeneity within low-grade neoplasms, and some were more aggressive in nature despite their low-grade histology. Consequently, there is potential for personalised and targeted treatment plans. For example, the poor-prognosis subtype had an overexpression of genes such as the *MET* proto-oncogene, known to activate multiple oncogenic pathways such as MAPK/ERK and P13K/AKT [93]. Consequently, there is an opportunity to use agents that target *MET* in this subset of patients. Rilotumumab, an inhibitor of hepatocyte growth factor/*MET* pathway was found to significantly increase OS in patients with overexpressed *MET* in gastric and oesophagogastric junction adenocarcinomas (ClinicalTrials.gov; ID: NCT00719550) [94]. If these molecular subtypes are to be used in a clinical setting to predict the outcomes of CRS/HIPEC and improve treatment efficacy, this warrants validation through a randomised-controlled clinical trial.

### Survival analysis in PMP and AMN

Prognosis has an important role in oncology for patients and clinicians to make informed decisions about the most appropriate treatment option and its outcome [95, 96]. Survival analyses are effective methods of testing prognosis, yet there is a paucity of data on the effect of gene mutations on the survival in PMP or AMN patients. Studies in this systematic review attempt to address this; however, there were conflicting conclusions on the effects of individual gene mutations on survival. We highlight the lack of consensus on the effect of individual genotypes on survival and suggest that molecular subtyping may be more effective in predicting survival as they have been proven to have prognostic value [31, 37, 53]. Within this remit, it may be useful to retrospectively map these molecular subtypes onto PMP datasets where transcriptomic data is readily available, to validate the prognostic value of the molecular subtypes.

### Strengths and limitations

The robustness of the methodology in this systematic review is a key strength. The abstract screening process was done in duplicate, and the two reviewers had a relatively high agreement rate of 99.91%. This is reflective of clear inclusion and exclusion criteria which were wide enough to capture the diversity of studies on this topic and precise enough to ensure that substantial conclusions may be drawn from the findings [97]. The search strategy was well-developed and included papers from 1995 to 2021 as the oldest study exploring the gene profile of AMN was published in 1996 [98]. Furthermore, there was a manual search of the reference list of Stein et al. [27] to account for any paper that may have been missed. Although the aims of the reviews were similar, there were notable differences in their study design compared to the current review. They only searched two databases and included studies up to 2016 whereas the current systematic review searched four databases and included studies up until 2020. This is important as there was an increase in publications on papers in this field between 2017 and 2021 (*n*=19), and studies published in 2020 introduced molecular subtypes and their role in prognosis [31, 37]. The risk of bias analysis identified that all studies in the review had a low risk of selection bias which indicated that their samples are accurate representations of the general population. The risk of bias analysis also provided an accurate assessment of the overall effect of the results which strengthened the conclusions drawn in this review.

Although this review yields valuable evidence about the genetic aberrations of PMP and AMN, there are some notable limitations. The sample sizes of older studies included in this review from 1996 to 2015 are relatively small (*n*=1) which may be due to the rarity and low incidence of PMP [1]. As a result, a small sample size may hinder the accuracy of statistical analyses due to low power [65]. Furthermore, NGS is more sensitive than Sanger sequencing and PCR and can identify more variants that may not be detectable by other techniques [99–101]. PMP has a low cellularity high mucin content, and therefore it is more challenging to perform NGS and Sanger sequencing since larger volumes of tumour cells are required [21]. Nonetheless, there is a risk that older studies (1996–2015) that used PCR may have failed to record key variations of the somatic mutations that were identified by NGS in more recent studies (2014–2020). Extreme heterogeneity in PMP histologies, for example the co-existence of high-grade with low-grade features in the same patient and the differences in histology between the primary (appendix) and metastatic (PMP) disease, may explain the differential prevalence rate of mutations seen. Moreover, the results cannot be averaged across studies due to the heterogeneity in study design, including sample size, tumour classification systems, tissue processing, and experimental methods used to investigate genetic aberrations. This highlights the need for greater standardisation of methods during validation so that the findings may be easily comparable, and conclusions may be more accurately translated into the clinical setting.

## Conclusions

In summary, this review identified the genetic aberrations in PMP and AMN. *KRAS*, *GNAS*, and *TP53* were the most frequently identified somatic mutations. Given the recent advances in clinical trials to directly target *KRAS* and *GNAS* mutations in other cancers, we propose a rationale to explore targeting *KRAS* G12C and G12D, and *GNAS* R201H and R201C in pre-clinical studies in PMP to produce preliminary results supporting a future clinical trial. Finally, given that molecular subtyping may be more effective in predicting survival, we encourage future clinical trials to complement the genotyping of PMP with transcriptomic analysis to validate and improve current molecular signatures and potentially develop more efficient clinical tools to predict survival and response to therapy.

## Supplementary information

ESM 1 : Table S1: Classification of mucinous epithelial neoplasia of the appendix, intra-abdominal mucin, and pseudomyxoma peritonei. (DOCX 19 kb)

ESM 2 : Table S2: Assessment of the quality and risk of bias of included studies. (DOCX 89 kb)

ESM 3 : Table S3: All gene mutations identified in this systematic review. (DOCX 105 kb)

ESM 4 : Table S4: Somatic mutations in *KRAS* G12D and predicted influence on protein function. (XLSX 11 kb)

ESM 5 : Figure S1: The RAS/Raf/MAP-Kinase Pathway. (DOCX 564 kb)

ESM 6 : Figure S2: *GNAS* in the cAMP-PKA pathway. (DOCX 662 kb)
