# Supplementary material for: The genetic profile and molecular subtypes of human pseudomyxoma peritonei and appendiceal mucinous neoplasms: a systematic review
Source: Cancer Metastasis Rev. 2023 Feb 1;42(1):335–59. doi: 10.1007/s10555-023-10088-0 (PMC10014681; doi:10.1007/s10555-023-10088-0)
Supplement: Supplementary file 1 — : Table S1: Classification of mucinous epithelial neoplasia of the appendix, intra-abdominal mucin, and pseudomyxoma peritonei. (DOCX 19 kb) [file 10555_2023_10088_MOESM1_ESM.docx]

**Supplementary Table S1:** Classification of mucinous epithelial neoplasia of the appendix, intra-abdominal mucin, and pseudomyxoma peritonei. Adapted from Carr *et al.*^2,11^.

| Classification of Mucinous Epithelial Neoplasia of the Appendix | |
| --- | --- |
| Classification | Histological Features |
| Low-grade appendiceal  mucinous neoplasm  (LAMN) | Mucinous neoplasm without infiltrative invasion but with any of the following:   - Loss of muscularis mucosae - Fibrosis of submucosa - “Pushing invasion” expansile or diverticulum-like growth - Dissection of acellular mucin in wall - Undulating or flattened epithelial growth - Rupture of appendix   Mucin and/or cells outside appendix |
| High-grade appendiceal  mucinous neoplasm (HAMN) | All the above as well as high-grade dysplastic features:   - Cribriform growth - Loss of polarity - Full thickness nuclear stratification - Enlarged nuclei - Markedly hyperchromatic or vesicular nucleoli - Atypical mitotic figures |
| Serrated polyp with or  without dysplasia (low- or high-grade) | Tumour with serrated features confined to the mucosa  Muscularis mucosae intact |
| Tubular, tubulovillous or  villous adenoma, low- or high-grade dysplasia | Adenoma resembling usual colorectal type  Confined to mucosa  Muscularis mucosae intact |
| Mucinous  adenocarcinoma- well, moderately, or poorly  differentiated | Mucinous neoplasm with infiltrative invasion |
| Adenocarcinoma- well,  moderately, or poorly  differentiated | Non-mucinous adenocarcinoma resembling usual  colorectal type |
| Poorly differentiated  (mucinous)  adenocarcinoma with  signet ring cells | Signet ring cells present in adenocarcinoma |
| Classification of Intra-Abdominal Mucin and Pseudomyxoma Peritonei | |
| Acellular mucin | Mucin within the peritoneal cavity without neoplastic  epithelial cells |
| Low-grade mucinous  carcinoma peritonei | Epithelial component typically scanty  Strips, gland-like structures, or small clusters of cells  Minimal cytological atypia  Not more than occasional (sporadic) mitosis  Invasion into underlying organs is generally of the “pushing” type |
| High-grade mucinous  carcinoma peritonei | Relatively more cellular  Cribriform growth  High-grade cytological atypia  Numerous mitoses  Destructive infiltrative invasion of underlying organs |
| High-grade mucinous  carcinoma peritonei with  signet ring cells | Any lesion with a component of signet ring cells i.e. round cells with intracytoplasmic mucin pushing the nucleus against the membrane  Degenerating cells within pools of mucin that mimic signet ring cells should be discounted |
