# Supplementary material for: The genetic profile and molecular subtypes of human pseudomyxoma peritonei and appendiceal mucinous neoplasms: a systematic review
Source: Cancer Metastasis Rev. 2023 Feb 1;42(1):335–59. doi: 10.1007/s10555-023-10088-0 (PMC10014681; doi:10.1007/s10555-023-10088-0)
Supplement: Supplementary file 2 — : Table S2: Assessment of the quality and risk of bias of included studies. (DOCX 89 kb) [file 10555_2023_10088_MOESM2_ESM.docx]

**Supplementary Table S2:** Assessment of the quality and risk of bias of included studies

| **Study** | **Was the confounding domain controlled appropriately?** | | **Bias due to confounding** | **Bias in selection of participants in the study**  **(selection bias)** | **Bias in classification of interventions**  **(information bias)** | **Bias due to missing data (selection bias)** | **Bias in measuring of outcomes (information bias)** | **Bias in selection of reported result (reporting bias)** | **Risk of bias judgement** |
| --- | --- | --- | --- | --- | --- | --- | --- | --- | --- |
|  | **Tumour grade** | **Origin of neoplasm** |  |  |  |  |  |  |  |
| Flatmark *et al*. 2021 | Yes | Yes | Low | N/A | N/A | N/A | N/A | N/A | Low |
| Moaven *et al*. 2020^68^ | No | Yes | Moderate | Low | Low | Low | Low | Moderate | Moderate |
| Liao *et al*. 2020^34^ | Yes | Yes | Low | N/A | N/A | N/A | N/A | N/A | Low |
| King *et al*. 2020^38^ | Yes | Yes | Low | N/A | N/A | N/A | N/A | N/A | Low |
| Yanai *et al*. 2020^39^ | Yes | Yes | Low | N/A | N/A | N/A | N/A | N/A | Low |
| Foster *et al*. 2020^40^ | No | Yes | Moderate | Low | Low | Low | Low | Low | Moderate |
| Lung *et al*. 2020^64^ | No | Yes | Moderate | Low | Low | Low | Low | Moderate | Moderate |
| Su *et al*.  2020^69^ | No | Yes | Moderate | Low | Low | Low | Low | Moderate | Moderate |
| Tsai *et al*. 2019^41^ | Yes | Yes | Low | N/A | N/A | N/A | N/A | N/A | Low |
| LaFramboise *et al*. 2019^35^ | Yes | Yes | Low | N/A | N/A | N/A | N/A | N/A | Low |
| Liu *et al*.  2019^66^ | No | Yes | Moderate | Low | Low | Low | Low | Low | Moderate |
| Tokunaga *et al*. 2019^42^ | No | Yes | Moderate | Low | Low | Low | Low | Moderate | Moderate |
| Zhu *et al*.  2019^43^ | Yes | Yes | Low | N/A | N/A | N/A | N/A | N/A | Low |
| Ang *et al*. 2018^70^ | Yes | Yes | Low | N/A | N/A | N/A | N/A | N/A | Low |
| Gleeson *et al.* 2018^8^ | Yes | Yes | Low | N/A | N/A | N/A | N/A | N/A | Low |
| Pengelly *et al.* 2018^67^ | Yes | Yes | Low | N/A | N/A | N/A | N/A | N/A | Low |
| Wen *et al.* 2018^44^ | Yes | Yes | Low | N/A | N/A | N/A | N/A | N/A | Low |
| Ang *et al.* 2017^71^ | No | Yes | Moderate | Low | Low | Low | Low | Low | Moderate |
| Matson *et al.* 2017^45^ | Yes | Yes | Low | N/A | N/A | N/A | N/A | N/A | Low |
| Saarinen *et al*. 2017^63^ | No | Yes | Moderate | Low | Low | Low | Low | Moderate | Moderate |
| Borazanci *et al.* 2016^46^ | No | Yes | Moderate | Low | Low | Low | Low | Moderate | Moderate |
| Pietrantonio *et al*. 2016^48^ | No | Yes | Moderate | Low | Low | Low | Low | Moderate | Moderate |
| Pietrantonio *et al*. 2016^47^ | No | Yes | Moderate | Low | Low | Low | Low | Low | Moderate |
| Levine *et al.* 2016^72^ | No | Yes | Moderate | Low | Low | Low | Low | Moderate | Moderate |
| Wu *et al.*  2015^62^ | No | Yes | Moderate | Low | Low | Low | Low | Moderate | Moderate |
| Noguchi *et al.* 2015^52^ | Yes | Yes | Low | N/A | N/A | N/A | N/A | N/A | Low |
| Hara *et al.* 2015^51^ | Yes | Yes | Low | N/A | N/A | N/A | N/A | N/A | Low |
| Roberts *et al.* 2015^73^ | Yes | Yes | Low | N/A | N/A | N/A | N/A | N/A | Low |
| Nummela *et al.* 2015^49^ | No | Yes | Moderate | Low | Low | Low | Low | Moderate | Moderate |
| Alakus *et al.* 2014^61^ | No | Yes | Moderate | Low | Low | Low | Low | Moderate | Moderate |
| Davison *et al.* 2014^53^ | No | Yes | Moderate | Low | Low | Low | Low | Moderate | Moderate |
| Liu *et al.*  2014^50^ | No | Yes | Moderate | Low | Low | Low | Low | Moderate | Moderate |
| Singhi *et al.* 2014^54^ | No | Yes | Moderate | Low | Low | Low | Low | Moderate | Moderate |
| Shetty *et al.* 2013^74^ | No | Yes | Moderate | Low | Low | Low | Low | Moderate | Moderate |
| Pulighe *et al.* 2013^75^ | No | Yes | Moderate | Low | Low | Low | Low | Low | Moderate |
| Nishikawa *et al.* 2013^65^ | No | Yes | Moderate | Low | Low | Low | Low | Moderate | Moderate |
| Zauber *et al.* 2011^55^ | Yes | Yes | Low | N/A | N/A | N/A | N/A | N/A | Low |
| Maheshwari *et al.* 2006^56^ | Yes | Yes | Low | N/A | N/A | N/A | N/A | N/A | Low |
| Sebastian *et al.* 2006^76^ | No | Yes | Moderate | Low | Low | Low | Low | Low | Moderate |
| Feltmate *et al.* 2005^60^ | No | Yes | Moderate | Low | Low | Low | Low | Low | Moderate |
| Maru *et al.* 2004^57^ | No | Yes | Moderate | Low | Low | Low | Low | Moderate | Moderate |
| O’Connell *et al.* 2002^77^ | Yes | Yes | Low | N/A | N/A | N/A | N/A | N/A | Low |
| Kabbani *et al.* 2002^58^ | No | Yes | Moderate | Low | Low | Low | Low | Moderate | Moderate |
| Shih *et al.* 2001^59^ | Yes | Yes | Low | N/A | N/A | N/A | N/A | N/A | Low |
| Szych *et al.* 1999^36^ | Yes | No | Moderate | Low | Low | Low | Low | Low | Moderate |
| Chuaqui *et al.* 1996^37^ | Yes | No | Moderate | Low | Low | Low | Low | Low | Moderate |
