# Supplementary material for: The genetic profile and molecular subtypes of human pseudomyxoma peritonei and appendiceal mucinous neoplasms: a systematic review
Source: Cancer Metastasis Rev. 2023 Feb 1;42(1):335–59. doi: 10.1007/s10555-023-10088-0 (PMC10014681; doi:10.1007/s10555-023-10088-0)
Supplement: Supplementary file 3 — : Table S3: All gene mutations identified in this systematic review. (DOCX 105 kb) [file 10555_2023_10088_MOESM3_ESM.docx]

**Supplementary Table S3: All gene mutations identified in this systematic review**

| **Study** | **All gene mutations identified** |
| --- | --- |
| Flatmark *et al.* 2021 | *GNAS*: 88%; R201H [N=16], R201C [N=6]  *KRAS*: 84%; G12D [N=12], G13D [N=3], G12V [N=5], both G12D and G13D [N=1] |
| Moaven *et al.* 2020^68^ | Genes associated with worse overall survival in LAMN: *PRSS3* (HR= 1.22, CI= 1.02-1.47); *EFNA1* (HR= 1.81, CI= 1.12-2.93)  Genes associated with worse overall survival in HAMN: *PUS1* (HR= 2.92, CI= 1.46-5.84); *GALNT4* (HR= 2.24, CI= 1.30-3.84); *CENPK* (HR= 1.89, CI= 1.15-3.10; *ERBB2* (HR= 1.88, CI= 1.05-3.36); *KIF23* (HR= 1.86, CI= 1.09-3.20); *UBE2C* (HR= 1.86, CI= 1.16-2.96); *CDKN3* (HR=1.78, CI= 1.06-2.97); *CENPA* (HR=1.77, CI= 1.20-2.60); *CXCL11* (HR=1.68, CI= 1.11-2.55); *MKI67* (HR=1.68, CI= 1.10-2.57); *CEP55* (HR=1.62, CI= 1.11-2.36); *NEK2* (HR=1.61, CI= 1.16-2.22); *GALNT3* (HR=1.60, CI= 1.14-2.25); *TRIM15* (HR=1.58, CI= 1.21-2.07); *NUF2* (HR=1.58, CI= 1.15-2.16); *EFNA1* (1.57, CI= 1.08-2.28); *FAM108C1* (HR=1.57, CI= 1.02-2.27); *TTK* (HR= 1.51, CI= 1.02-2.23); *FERMT1* (HR=1.50, CI= 1.02-2.21); *TPX2* (HR=1.50, CI= 1.02-2.20); *F2RL1* (HR= 1.48, CI= 1.04-2.11); *CEACAM1* (HR=1.48, CI= 1.04-2.11); *ARL14* (HR=1.46, CI= 1.09-1.95); *FOXQ1* (HR=1.33, CI=1.04-1.70); *Clorf106* (HR=1.31, CI= 1.05-1.62); *GCNT3* (HR= 1.29, CI= 1.03-1.61); *DUOX2* (HR=1.29, CI= 1.09-1.52); *MUC13* (HR=1.28, CI= 1.01-1.60); *AGR2* (HR=1.27, CI= 1.04-1.55); *MUC4* (HR=1.26, CI= 1.05-1.50); *SLC44A4* (HR= 1.21, CI= 1.02-1.44); *TMPRSS4* (HR=1.20, CI= 1.01-1.42); *CLCA1* (HR= 1.14, CI= 1.02-1.27)  Genes associated with better overall survival in HAMN: *IGHA1* (HR= 0.81, CI= 0.67-0.97); *KLRG1* (HR= 0.74, CI= 0.56-0.97); *TPSAB1* (HR= 0.62, CI= 0.42-0.93); *TRA* (HR= 0.56, CI= 0.37-0.84); *TSC22D3* (HR= 0.45, CI= 0.21-0.98) |
| Liao *et al.* 2020^34^ | LAMN: *KRAS*: 100%, c.34G>T *p.G12C* [N=1], c.35G>A *p.G12D* [N=5], c.35G>T *p.G12V* [N=2]  *GNAS*: 63%, c.601C>T *p.R201C* [N=4], c.602G>A *p.R201H* [N=2].  *KRAS* and *GNAS* co-occurrence in 63%  HAMN: *KRAS*: 100%, c.35G>A *p.G12D* [N=6], c.35G>T *p.G12V* [N=2]  *GNAS*: 56%, c.601C>T *p.R201C* [N=1], c.602G>A *p.R201H* [N=4]  *TP53*: 44%, c.531_533del p.H178del [N=1], c.637C>T *p.R213** [N=1], c.817C>T *p.R273C*, c.856G>A *p.E286K*  *ATM*: 22%, c.9022C>T *p.R3008C* [N=1], c.9023G>A *p.R3008H* [N=1]  MAC: *KRAS*: 70%, c.183A>C *p.Q61H* [N=1], c.35G>A *p.G12D* [N=2], c.35G>T *p.G12V* [N=3], c.40G>A *p.V14I* [N=1];  *GNAS*: 10%, c.183A>C *p.Q61H* [N=1];  *TP53*: 50%, c.1024C>T *p.R342** [N=1], c.527G>T *p.C176F* [N=1], c.584T>C *p.I195T* [N=1], c.712T>C *p.C238R* [N=1], c.817C>T *p.R273C* [N=1];  *PIK3CA*: 20%; *APC*: 10%; *FBXW7*: 10%; *PTEN*: 10%; *SMAD4*: 10% |
| King *et al.* 2020^38^ | Mother and daughter: pathogenic *RAD51C* (*R319** c.955C>T) and pathogenic *KRAS* (*G12D*). Variants of uncertain significance- *FH* (*K477_N478insK*) and *TSCI* (*H732Y*)  Mother only: pathogenic *GNAS* (*R201H*), pathogenic *DIS3* (*D458N*), two variants of uncertain significance *AXIN1* (*R484C*) and *PRDM1* (*A711T*)  Daughter only: pathogenic *GNAS* (*R201C*), pathogenic *RB1* (*R251**), pathogenic *SMAD4* (*R361H*). 5 variants of uncertain significance *CREBBP* (*P1010L*), *FANCA* (*L379V*), *MED12* (*Q2119_Q2120insHQQQ*), *PIK3C2B* (*R1118C*), *SMARCA4* (*A1536S*) |
| Yanai *et al.* 2020^39^ | LAMN (N=34): *KRAS*: 53% (N=18), *G12D* (N=8), *G12F* (N=1), *G12V* (N=3), *G13D* (4), *E61H* (N=1); *GNAS*: 15% (N=5), *R201C* (N=1), *R201H* (N=3), *R201S* (N=1); *KRAS+GNAS*: 15% (N=5); *RNF43*: 21% (N=7), *S643T* (N=1), *I46V* and *T58S* (N=1), *A70Y* (N=1), *D56G* (N=1), *T350I* (N=1), *L63F* and *c.676Cfs* (N=1), *R253G* and *P307L* (N=1); *TP53*: 18% (N=6), *S127F* (N=1), *G267R* (N=1), *R306** (N=1), *R213** (N=1), *Q192** (N=1), *S99F* and *G245V* (N=1); LOH *TP53*: 46%; *RET*: 3% (N=1), *p.Y900*;* *GNAQ*: 3% (N=1), *T224N*; *SMAD4*: 3% (N=1), *Q256L* Specific gene mutations in PMP (6): *KRAS*: 67% (N=4), *G12D* (N=3), *G12F* (N=1); *GNAS*: 17% (N=1), *R201H* (N=1); *RNF43*: 33% (N=2), *S643T* (N=1), *T350I* (N=1);  *TP53*: 17% (N=1), *S99F* and *G245V* (N=1)  HAMN without PMP (N=1) *RNF43*: *K171R*  HAMN with PMP (N=7): *KRAS*: 38% (N=3) *G12D* (N=1), *G12A* (N=1), *G12V* (N=1); *GNAS*: 38% (N=3), *R201C* (N=1), *R201H* (N=2); *KRAS+GNAS*: 13% (N=1); *RNF43*: 13% (N=1), *Q679** and *K171R*; *TP53*: 25% (N=2), *Q43fs* (N=1), *R337C* (N=1); LOH *TP53*: 17%; *SMAD4*: 13% (N=1), *Q256L*; *SMARCB1*: 13% (N=1), *R377H*; *EGFR*: 13% (N=1), *G873R*  MAC (9): *KRAS*: 44% (N=4), *G12D* (N=1), *G12V* (N=2), *G13D* (N=1); *GNAS*: 33% (N=3), *R201H* (N=2), *R201S* (N=1); *KRAS+GNAS*: 22% (N=2); *RNF43*: 44% (N=4), *I46V* and *A70Y* (N=1), *S475F* and *V509A* (N=1), *P378S* and *A672T* (N=1), *c.I47V* and *M179I* (N=1); *TP53*: 22% (N=2), *R249G* (N=1), *G266E* (N=1); LOH *TP53*: 67%; *APC*: 11% (N=1), *A1582P*; *SMAD4*: 22% (N=2), *R361C* (N=2); *RB1*: 11% (N=1), *L670P*; *ERBB2*: 11% (N=1), *V842I*; *GNAQ*: 11% (N=1), *T224N* Specific gene mutation in PMP (1): *TP53*: *G266E* |
| Foster *et al.* 2020^40^ | Bold are actionable mutations in these genes: *KRAS*: 85%, ***G12D* (6), *G12S*, *G12C*, *G12V*, *K147E*, *A146T***; *GNAS*: 77%, *R844C* (5), *R844H* (4), *Q870H* ; *TP53*: 31% ***R282W*, *R273C*, *R249S***, *E171K*, *R175H*; *PIK3CA*: 8%, ***Q546K***.  All *GNAS* mutations occurred in conjunction with a *KRAS* mutation |
| Lung *et al.* 2020^64^ | Nonsense: *REEP5* Missense: *EXOG5, REANBP2, RANBP6, TNFRSF1B* |
| Su *et al.* 2020^69^ | Identification of 3 molecular subtypes using a 17-gene signature Immune-enriched group: increased expression of IL23A, *CD37, KLRF1, MAFB, PRF1, TRA, TRBC1* Oncogene-enriched group: increased expression of *CLDN3, CLDN4, ELF3, EPCAM, GPX2, KRT20, LGALS4, PHGR1, ESRP1, SPINK1* Mixed-group showed mixed expression of the 17-gene signature |
| Tsai *et al.* 2019^41^ | LAMN (17): *KRAS*: 88%, *p.G12D* (N=11), *p.G12V* (N=4), *p.G13D* (N=1); *GNAS*: 41%, *p.R201C* (N=2), *p.R201H* (N=5); *RNF43*: 12%, *p.W238fs* (N=1), *p.P270fs* (N=1); *CTNNB1*: 6%, *p.S37C* (N=1); *APC*: 6%, *p.T1556fs* (N=1)  LAMN + PMP (6): *KRAS*: 100%, *p.G12D* (N=2), *p.G12V* (N=4); *GNAS*: 83%, *p.R201C* (N=2), *p.R201H* (N=3)  HAMN (N=3): *KRAS*: 100%, *p.G12D* (N=3); *TP53*: 33%, *p.P190L* (N=1); *APC*: 33%, *p.302** (N=1); *RNF43*: 33%, *p.ARG145** (N=1)  HAMN with PMP (N=2): *KRAS*: 100%, *p.G12C* (N=1), *p.G12D* (N=1); *GNAS*: 50%, *p.R201H* (N=1)  High grade mucinous carcinoma (N=2): *KRAS*: 100%, *p.G12D* (N=1), *p.G12V* (N=1); *TP53*: 50%, *p.R273G* (N=1)  High grade mucinous carcinoma + PMP (N=1): *KRAS*: 100%, *p.G12V*, *TP53 p.R273G* (N=1) |
| LaFramboise *et al.* 2019^35^ | *KRAS*: 100%, *G12D* (4), *G12V* (5), *G12A* (1) *GNAS*: 40%, *R844C* (1), *S187A* (1), *R844H* (2) *TP53*: 30% [All high-grade], *H179D* (1), *R175H* (1), *R248W* (1) *MYB*: 30% [All high-grade], *P512S* (1), *P453T* (1), *S492F* (1) Only seen in low-grade neoplasms: *APC*: 40%; *SH2D1A*: 30%; *TCF7L2*: 30%; *AR*: 30%  Only seen in high-grade neoplasms (MACs): *MYC*: 50%; *DAXX*: 40%; *PIM1*: 40%; *POU5F1*: 30%; *GATA2*: 30%  Additional mutations: *NFE2L2, CCNE1, KFL6, MEN1, HSP90AB1* |
| Liu *et al.* 2019^66^ | *KRAS*: missense, p.Gly12Asp (exon 2) *KDR/VEGFR-2*: missense, p.Gln472His (exon 11) *FGFR1*: missense, p.Arg281Gln (exon 8) *FGFR2*: missense, p.Lys296Arg (exon 7) *FGFR3*: missense, p.Thr654Ser (exon 14) Additional mutations: *TP53, ERBB2, KIT, GNA11, JAK3, AKT1, ATM, CSFIR, FLT3, KDR, NOTCH1, PIK3CA, PET, SMARCB1, SMO, STK11* |
| Tokunaga *et al.* 2019^42^ | PMP: *KRAS*: 74%; *GNAS*: 63%; *TP53*: 23%; *SMAD4*: 15%; *RNF43*: 6%; *APC*: 2%; *PIK3CA*: 2%  MAC: *KRAS*: 64%; *GNAS*: 25%; *TP53*: 57%; *SMAD4*: 20%; *RNF43*: 7%; *APC*: 16%; *PIK3CA*: 7%; *BRAF*: 9%; *ARID1A* 15%  Microsatellite instability: PMP- 0 (microsatellite stable), MAC- 2.4% (low)  Tumor mutational burden: PMP- 0, MAC- 2.4%  Programmed death ligand 1: PMP- 1.6%, MAC- 2.3% |
| Zhu *et al.* 2018^43^ | LAMN: *KRAS*: 90%; *GNAS*: 62%; *TP53*: 10%; *SMAD4*: 5%; *NRAS*: 5%; *IDH2*: 5%; *CDKN2A*: 5%  *KRAS* and *GNAS* co-occurrence: 52%   Moderately differentiated MAC: *KRAS*: 86%; *GNAS*: 67%; *TP53*: 29%; *SMAD4*: 19%; *PIK3CA*: 19%; *BRAF*: 10%; *RB1*: 5%; *CTNNB1*: 5%; *PTPN11*: 5%; *KDR*: 10%; *KIT*: 5%; *PTEN*: 10%  *KRAS* and *GNAS* co-occurrence: 29%  Poorly differentiated MAC (N=26): *KRAS*: 46%; *GNAS*: 12%; *TP53*: 27%; *SMAD4*: 12%; *PIK3CA*: 8%; *BRAF*: 8%; *CDKN2A*: 4%; *APC*: 4%; *MET*: 4%; *CDH1*: 4%; *ABL*: 4%  *KRAS* and *GNAS* co-occurrence: 8% |
| Ang *et al.* 2018^70^ | PMP: *KRAS* (81%: 40% *G12D*, 35% *G12V*, 5% *G12C*, 5% *G13D*), *GNAS* (72%: 25% *R201H*, 50% *R201C*), *SMAD4* (11%), *APC* (2%), *ARID1A* (6%), *TP53* (7%, missense), *ERBB2* (4%)  MAC: *KRAS* (77%: 45% *G12D*, 25% *G12V*, 5% *G12C*, 5% *G13D* 5% other), *GNAS* (52%: 20% *R201C*, 30% *R201H*, 5% other), *SMAD4* (23%), *APC* (6%), *ARID1A* (8%), *TP53* (33%: 20% missense, 5% nonsense 4% frameshift, 2% splice), *RB1* (2%), *ERBB2* (3%), *MSI-H* (1.4%) |
| Gleeson *et al*. 2018^8^ | *KRAS*: 81%; *GNAS*: 74%; *SMAD4*: 16%; *ATM*: 16%; *APC*: 11%; *cMET*: 11%; *PIK3CA*: 10%; *BRAF*: 8%; *TP53*: 5%; *MLH1*: 5%;  *NRAS*: 5% Co-mutation rate for *GNAS* and *KRAS*: 87%  *KRAS* variants: *G12D* (57%), *G12V* (43%) *GNAS* variants: *R201H* (54%), *R201C* (46%) *SMAD4*: (*G386D, R496H, S474X*); *ATM*: (*R2443Q*, *A1309T*); *BRAF*: (*V600E, D594G*); *PIK3CA*: (*E545K, E545G*); *cMET*: (*A319T, T1010I*); *APC*: (*L1129S, T1556fs*); *TP53*: (*L194R*); *NRAS*: (*Q61R*); *MLH1*: (*S406N*)  Gene mutations by histologic categories: *KRAS*: total= 81% (N=25), DPAM/PMCA-I 85% (N=23), PMCA 50% (N=2) *GNAS*: total= 74% (N=14), DPAM/PMCA-I 87% (N=13), PMCA 25% (N=1) *APC*: total= 11% (N=2), DPAM/PMCA-I 13% (N=2) *ATM*: total= 16% (N=3), DPAM/PMCA-I 13% (N=2), PMCA 25% (N=1) *BRAF*: total= 8% (N=2), DPAM/PMCA-I 10% (N=2) *cMET*: total= 10% (N=2), DPAM/PMCA-I 13% (N=2) *CSF1R*: total = 5% (N=1), DPAM/PMCA-I 7% (N=1) *MLH1*: total = 5% (N=1), DPAM/PMCA-I 7% (N=1) *NRAS*: total = 5% (N=1), PMCA 25% (N=1) *PIK3CA*: total = 10% (N=2), DPAM/PMCA-I 12% (N=2) *SMAD4*: total = 16% (N=3), DPAM/PMCA-I 13% (N=2), PMCA 25% (N=1) *TP53*: total = 5% (N=1), PMCA 25% (N=1) |
| Pengelly *et al.* 2018^67^ | *KRAS* (100%): *p.G12V* (20%, nonsynonymous, region of LOH on chromosome 12)  *GNAS* (100%): *p.R186H* (40%), nonsynonymous, region of LOH on chromosome 20)  *APC* (20%), *FGFR2* (20%), *FAT4* (20%)  LOH on chromosome 8: *EZH1* (20%, nonsynonymous), *RNF43* (20%, stopgain), *RAB37* (20%, nonsynonymous)  LOH on chromosome 12: *ERGIC2* (20%, nonsynonymous)  LOH on chromosome 18: *SMAD2* (20%, stopgain)  LOH on chromosome 20 (nonsynonymous): *ST18* (20%), *C8orf34* (20%), *COL22A1* (20%), *FERMT1* (20%), *C20orf203* (20%), *RGS19* (20%) |
| Wen et al. 2018^44^ | *KRAS p.G12D*, variant allele frequency 30% *GNAS p.R201H*, variant allele frequency 28%, tumor fraction 50% |
| Ang et al. 2017^71^ | *GNAS R201H* (activating mutation) |
| Matson et al. 2017^45^ | *KRAS* (c.35G>T p.G12V)  *GNAS* (c.602G>A p.R201H) |
| Saarinen et al. 2017^63^ | *KRAS*: 100% (N=9) *GNAS*: 56% (N=5)  *OXTR*: 11% (N=1); *EDNRA*: 11% (N=1); *PRKAR1A*: 11% (N=1); *RYR2*: 11% (N=1); *GUSB*: 11% (N=1); *GRIA2*: 11% (N=1); *CNGB1*: 11% (N=1); *LTBP1*: 11% (N=1); *THBS1*: 11% (N=1); *TGFBR2*: 22% (N=2); *PPP2R1B*: 11% (N=1); *ACVR1B*: 11% (N=1), *DUSP22*: 11% (N=1) |
| Borazanci et al. 2016^46^ | MAC: *ABL1*: 1.2%; *AKT1*: 1.2%; *APC*: 6.8%; *ATM*: 6.9%; *BRAF*: 1.7%; *cKIT*: 2%; *cMET*: 2.3%; *ERBB2*: 1.2%; *FBXW7*: 5.8%; *GNAS*: 33.8%;  *JAK3*: 2.3%; *KRAS*: 65%; *MLH1*: 2.3%; *PIK3CA*: 6.1%; *PTEN*: 1.2%; *SMAD4*: 15.1%, *SMO*: 1.3%; *STK11*: 1.3%; *TP53*: 24.4%  PMP: *GNAS*: 57.1%; *KRAS*: 83.3%; *PIK3CA*: 11.1%; *SMAD4*: 14.3%  Mucinous cystadenocarcinoma: *KRAS*: 100% |
| Pietrantonio et al. 2016^48^ | *KRAS*: 72% (N=29), *G12V* (N=6), *G12D* (N=20), *G13D* (N=2), *G12C* (N=1)  *GNAS*: 52% (N=21) *R201C* (N=5), *R201H* (N=15), *Q227STOP* (N=1) *GNAS* mutations were associated with *KRAS* mutations (P=0.002) *PIK3CA*: 7.5% (N=3), *H1047R* (N=1), *E545K* (N=1), *N345Y* (N=1)  *TP53*: 10% (N=4), *Y220C* (N=1), *P151S* (N=1), *R248W* (N=1), *Q192Stop* (N=1)  *AKT*: 2.5% (N=1) T172I; *LKB1*: 2.5% (N=1) *P319S*; *FGFR3*: 2.5% (N=1) A257V; *PDGFRA*: 2.5% (N=1) *R558C*;  *CTNNB1*: 2.5% (N=1) *D32N*; *SMO*: 2.5% (N=1) *E208K*; *SMAD4*: 2.5% (N=1) *R135STOP*; *CDH1*: 2.5% (N=1), *P373L*  Mutant allelic fraction frequencies: *KRAS*: 9% (range 1-57%) and <10% cut-off in 16 cases *GNAS*: 11% (range 4-57%) and <10% cut-off in 9 cases |
| Pietrantonio et al. 2016^47^ | *KRAS*: 93% (N=14)- *G12C* (N=1), *G12D* (N=8), *G12V* (N=3), *G13D* (N=2) *GNAS*: 60% (N=9)- *R201H* (N=8), *R201C* (N=1) *TP53*: 20% (N=3)- *R248W* (N=1), *R273H* (N=1), *C238F* (N=1) *HNF1A*: 7% (N=1)- *R278W* *FGFR3*: 7% (N=1)- *A257V* *LKB1*: 7% (N=1)- *P319S* *FGFR3* and *LKB1* occur in the same case |
| Levine et al. 2016^72^ | Increased expression of 139 gene signature associated with poor patient outcome and characteristic of poor prognosis subtype Enrichment of genes involved in p53 mutation, *AKT* activation, *HER2* overexpression, glycolysis, epithelial to mesenchymal transition, and *E2F* target in poor prognosis subtype Increased expression of *CD24*, *PROM1*, *EPCAM* (cancer stem cell markers) in poor prognosis subtype |
| Wu et al. 2015^62^ | *miR-1* and *miR-4328* significantly downregulated in most of the mucinous cystadenocarcinoma samples compared to mucinous cystadenoma *miR-200b*, *miR-200c, miR-223, miR-451, miR-21* significantly increased in mucinous adenocarcinoma compared to the mucinous adenoma |
| Noguchi et al. 2015^52^ | *KRAS*: 78% (missense, *p.G12D* [N=6], *p.G12V* [N=6], *p.G12S* [N=1], *p.G13D* [N=1]); *GNAS*: 44% (missense, *p.R201H* *p.R201C*), *TP53*: 22% (missense, *p.R175H*, *p.P250L, p.G226R, p.R273C*); *SMAD4*: 17% (deletion *p.185QfsX16* [N=1], insertion *p.L529fs* [N=1], missense *p.C499R* [N=1]); *AKT1*: 6% (missense, *p.E17K*); *NRAS*: 6% (missense, *p.Q61K*); *PIK3CA*: 6% (missense, *p.M1043V*); *PDGFRA*: 6% (missense, *p.P553L*); *RET*: 6% (missense, *p.E884V*); *VHL*: 6% (missense, *p.G144R*)  DPAM: *KRAS*: 80%; *GNAS*: 50%; *SMAD4*: 20%; *RET*: 10%; *VHL*: 10%  PMCA: *KRAS*: 75%; *GNAS*: 38%; *TP53*: 38%; *PIK3CA*: 13%; *AKT1*: 13%; *SMAD4*: 13%; *NRAS*: 13%; *PGDFRA*: 13%  Nucleotides: *KRAS*: *G12S* c.34G>A, *G12D* c.35G>A, *G12V* c.35G>T, *G13D* c.38G>A; *GNAS*: *R201C* c.GO1C>T, *R201H* c.602G>A; *TP53*: *R175H* c.524G>A, *P250L* c.749C>T, *G266R* c.796G>C, *R273C* c.817C>T; *SMAD4*: *P185QfsX16* c.553_556delCCAC, *L529fs* c.1586_1587insA, *C499R* c.1495T>C; *AKT*: *E17K* c.49G>A; *NRAS*: *Q61K* c.181C>A; *PDGFRA*: *P553L* c.1658C>T; *PIK3CA*: *M1043V* c.3127A>G; *RET*: *E884V* c.2651A>T; *VHL*: *G144R* c.430G>A |
| Hara et al. 2015^51^ | LAMN:  *GNAS*: 18% (*R201C*, *R201H*); *KRAS*: 27% (*Q25X*, *D30Y*, *G12S*); *CTNNB1*: 18% (*T40A*) *TP53*: 27% (*C141R*, *D184G*, *P250S*)  MAC:  *GNAS*: 20% (*Q227X*, nonsense mutation, not activating); *KRAS*: 60% (*G15D*, *G12D*, *G12V*)  *TP53*: 60% (*D206G*, [case13: *A161V*, *E171K*, *G245D* and *A275V*], and [case15: *R247G* and *S260T*]), observed only in high grade areas: *G245D* and *R249G* |
| Roberts et al. 2015^73^ | Upregulated: *SLC16A4*, *DSC3*, *ALDOB*, *EPHX4*, *ARHGAP24* Downregulated: *MS4A12*, *TMIGD1*, *Caspase-5* |
| Nummela et al. 2015^49^ | *KRAS*: 100%- *p.G12D* (N=12, 6LG, 6HG), *p.G12C* (N=3, 2LG, 1HG), *p.G13D* (N=4, 1LG, 1HG) *GNAS*: 63.2%- *p.R201C* (N=5, 1 LG, 1HG), *p.R201H* (N=7, 4LG, 3HG) *SMAD4*: 15.8%- *p.G386D* (N=1, HG), *p.R361H* (N=1, HG), c.1067_1068delCT (N=1, HG) *AKT1*: 5.3% (N=1, HG)- *p.E17K* *ATM*: 5.3% (N=1, HG)- *p.L1327* *PIK3CA*: 5.3% (N=1,LG)- *p.E545K* *TP53*: 5.3% (N=1, HG)- *p.N239D* |
| Alakus et al. 2014^61^ | 19 MNA patients validated by PCR: *KRAS* 89% (gain of function), *GNAS* 79% (14 gain of function, 1 unknown), *SMAD2* 11% (loss of function) 10 MNA exome sequencing:  *KRAS*: 100% (gain of function, *G12V*=7, *G12D*=1, *A146T*=1; co-occurrence of *G12V* and *G12D*)  *GNAS*: 90% (gain of function, *R201C*=7, *R201H*=1, *Q227H*=1)  *PRKACA*: 10% (gain of function)  *SMAD2*: 20% (loss of function, Ser465, homozygous due to LoH chromosome 18)  *SMAD3*: 10% (unknown function)  *SMAD4*: 30% (unknown function)  *TGFBR1*: 10% (loss of function, homozygous)  *TGFBR2*: 10% (loss of function, homozygous)  *FAT4*: 30% (unknown function)  *CTNNB1*: 10% (gain of function)  *TP53*: 10% (loss of function) |
| Davison et al. 2014^53^ | LOH of *SMAD4*: Total: 17% LAMN: 5% Moderately differentiated MAC: 27% Poorly differentiated MAC: 22% |
| Liu et al. 2014^50^ | LAMN: *KRAS*: 25%, (c.34G>T, *p.G12V* [N=1]; c.35G>A, *p.G12D* [N=5]; c.38G>A, *p.G13D* [N=1]; c.183A>C, *p.Q61H* [N=1]) *GNAS*: 24%, (c.2531G>A, *p.R844H* [N=2]; c.2530C>T, *p.R844C* [N=6]) *KRAS* and *GNAS* co-occurrence in 75% Additional mutations: *AKT1*: 3.75%; *APC*: 15%; *JAK3*: 3.75%; *MET*: 6%; *PIK3CA*: 3.75%; *RB1*: 6%; *STK11*: 3.75%  Well-differentiated MAC with PMP: *KRAS*: 43%, (c.35G>A, *p.G12D*; c.35G>T, *p.G12V*; c.34G>T, *p.G12C*) Additional mutations: *TP53*: 14%; *GNAS*: 22%; *RB1*: 7%  MAC: *KRAS* (c.37G>A, *p.G13R*)  *IDH1* (c.394C>A, *p.R132S*) |
| Singhi et al. 2014^54^ | *GNAS*:  31% (N=17), *p.R201H* c.6002G>A (N=10), *p.R201C* c.602C>T (N=7) LAMN: 35%; Moderately differentiated MAC: 37%; Poorly differentiated MAC: 15%  *KRAS*: 40% (N=22), *p.G12D* c.35G>A (N=14), *p.G12V* c.35G>T (N=7), *p.G13D* c.38G>A (N=1) |
| Shetty et al. 2013^74^ | *KRAS*: 57.8% Codon 12: 88.6% (N=31): Gly>Asp (N=20, 10LG, 10HG); Gly>Val (N=8, 2LG, 6HG); Gly>Cys (N=3, 1LG, 2HG)  Codon 13: 11.4% (N=4): Gly>Asp (N=2, 2HG); Gly>Arg (N=1, 1LG); Gly>Cys (N=1, 1HG) |
| Pulighe et al. 2013^75^ | *PIK3CA*: p.S553fs*7 in exon 9 |
| Nishikawa et al. 2013^65^ | LAMN: *GNAS*: 50% (N=16)- p.*R201S* (N=1 nucleotide c.601C>A), *p.R201C* (N=6 c.601C>T), *p.R201H* (N=7, c.602G>A),  BOTH p.R201C and *p.R201H* (N=2 c.601C>T [*R201C*], c.602G>A [*R201H*]) *KRAS*: 94% (N=30)- *p.G12C* (N=1, c.34G>T), *p.G12D* (N=13, c.35G>A), *p.G12A* (N=1, c.35G>C), *p.G12V* (N=11, c.35G>T),  *p.G13D* (N=4, c.38G>A)  MAC: *KRAS*: 100% (N=3) *p.G12S* (c.34G>A) |
| Zauber et al. 2011^55^ | *KRAS*: 100% Codon 12: 90% (N=28)- GGT>TGT (N=1), GGT>GAT (N=13), GGT>GTT (N=13), GGT>GCT (N=1) Codon 13: 10% (N=3)- GGC>GAC All were microsatellite stable |
| Maheshwari et al. 2006^56^ | Fractional Mutation Rate (calculated by using 6 markers and KRAS mutational changes) DPAM (N=6): .00 (N=5); .17 (N=1) PMCA-I (N=7): .00 (N=4); .33 (N=1); .43 (N=1); .86 (N=1) PMCA (N=10): .67 (N=2); .71 (N=2); .80 (N=1); .83 (N=2); .87 (N=1); 1.00 (N=2) |
| Sebastian et al. 2006^76^ | *KRAS*: point mutation G12S |
| Feltmate et al. 2005^60^ | LOH in percentages (frequency) 1p13.3-22.3 63%; 1p21.1-22.3 33%; 1p36.2-36.3 25%; 1q31.2-31.3 14%; 1q43-44 50%; 2q24.1-24.3 44%; 3p25 17%; 3q24 56%; 3q26.32 75%; 4q13 40%; 4q24 50%; 5q23.2 63%; 5q32-33.1 14%; 6p21.2 17%; 6q21 67%; 6q24 75%; 6q25.2 75%; 8p11.2-12 57%; 8p23-22 45%; 9p22.1 22%; 9p24.3 88%; 9q22.31 50%; 9q33 17%; 9q34.3 38%; 10p15 50%; 11q12 38%; 11q25 29%; 13q21 50%; 13q31.3 60%; 14q11.2 50%; 14q23.2 36%; 14q32 33%; 15q26.1 33%; 16p13.2 50%; 16q24.1 50%; 17p12-11.2 50%; 17p13.3 33%; 18p11.32 50%; 18q23 71%; 19q13.2 25%; 20p12.3 29%; 20q13.1 43%; 21q11.2 56%; 21q21.3 60%; Xp21 33%; Xp11.4 40%; Xq27 50%; Xq 28 100% |
| Maru et al. 2004^57^ | Allelic loss of chromosome 18q: 53.8% *SMAD4*: 21.4%, missense mutation in exon 8 [N=1] and exon 11 [N=1], 30 bp deletion involving the stop codon [N=1] |
| O’Connell et al. 2002^77^ | PMP:  *MUC2*: 95% (DPAM: 98%, PMCA-I: 90%, PMCA: 80%)  *MUC5AC*: 90% (DPAM: 96%, PMCA-I: 80%, PMCA: 70%)  Solitary mucinous tumours of the appendix:  *MUC2*: 100%  *MUC5AC*: 100% |
| Kabbani et al. 2002^58^ | *KRAS*: 50% |
| Shih et al. 2001^59^ | *KRAS*: PMP- Codon 12 GGT>GTT Mucinous adenoma- Codon 12 GGT>GAT  *APC* LOH only in the mucinous adenoma |
| Szych et al. 1999^36^ | PMP:  *KRAS*:100% LOH analysis of polymorphic microsatellite loci: D5S592 (5q22.1): 13%; D6S474 (6q16.3-22.33): 7%; D6S1027 (6q27): 7%  Mucinous adenomas:  *KRAS*: 69% D5S592: 27%; D6S474: 7%; D6S1027: 13%; D17S1303 (17p13.1-13.3): 21%; D18S51 (18q21.33): 33%;  D18S499 (18q21.32-21.33): 19% |
| Chuaqui et al. 1996^37^ | Case 1 (invasive carcinoma): LOH at 3p Case 2 (non-invasive carcinoma): LOH at 3p Case 4 (invasive carcinoma): LOH at 17q Case 10 (non-invasive carcinoma): LOH at 5q |
