# Supplementary material for: The genetic profile and molecular subtypes of human pseudomyxoma peritonei and appendiceal mucinous neoplasms: a systematic review
Source: Cancer Metastasis Rev. 2023 Feb 1;42(1):335–59. doi: 10.1007/s10555-023-10088-0 (PMC10014681; doi:10.1007/s10555-023-10088-0)
Supplement: Supplementary file 5 — : Figure S1: The RAS/Raf/MAP-Kinase Pathway. (DOCX 564 kb) [file 10555_2023_10088_MOESM5_ESM.docx]

**Supplementary Figure S1: The RAS/Raf/MAP-Kinase Pathway**


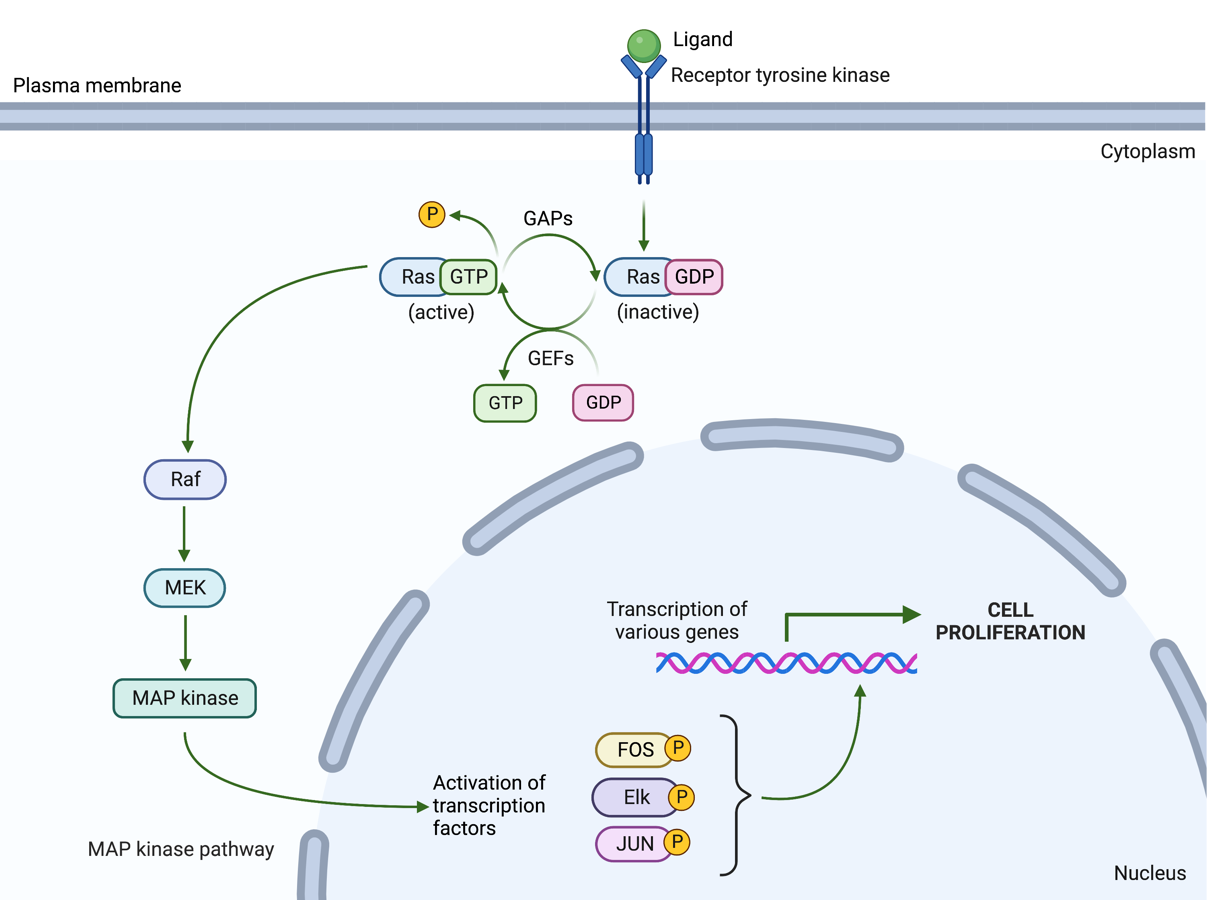


**A**

**B**


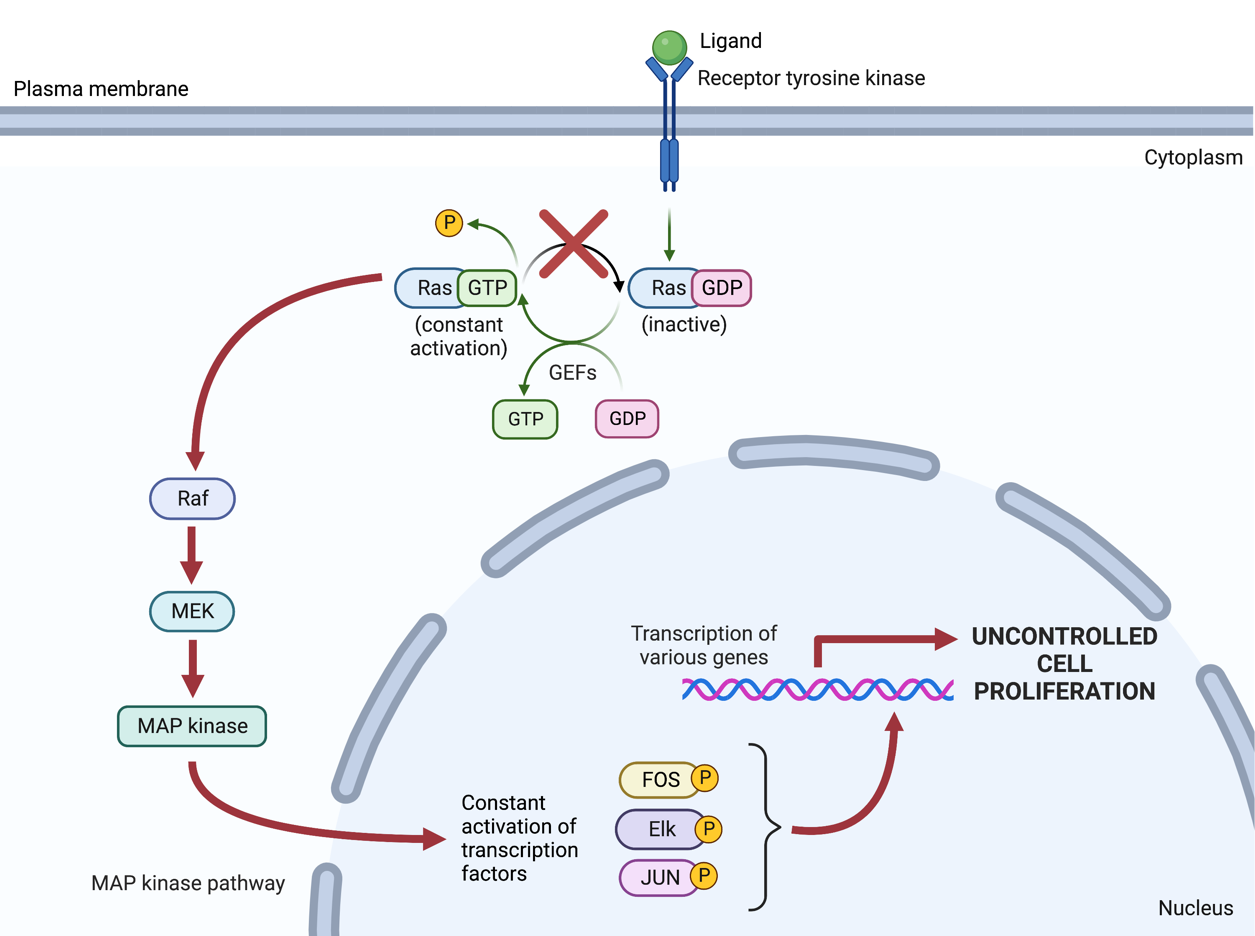


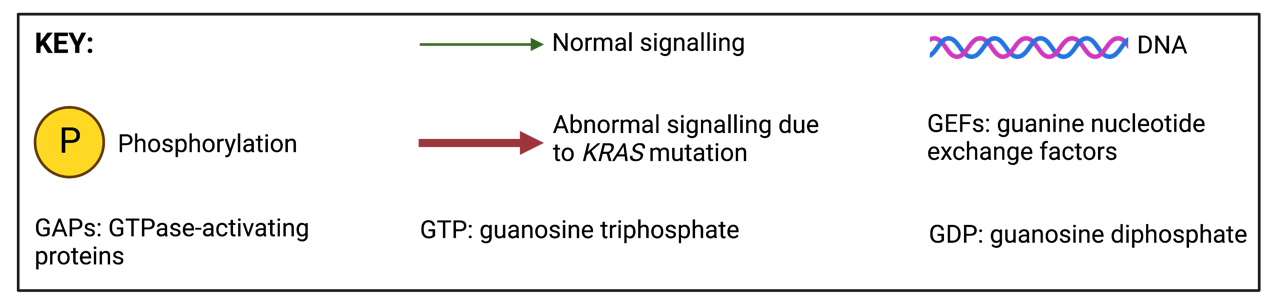


**Supplementary Figure 1: The RAS/Raf/MAP-Kinase pathway. (A)** When an extracellular ligand binds to the protein tyrosine kinase receptor, this activates RAS by stimulating guanine nucleotide exchange factors (GEFs) which are involved in converting guanosine diphosphate (GDP) to guanosine triphosphate (GTP). The *KRAS* gene codes for the RAS protein which relays extracellular signals to the nucleus by binding to RAF, activating downstream signalling of MEK and MAP kinase. The activated form of MAP kinase activates transcription factors (such as Elk, FOS, and JUN) through phosphorylation which lead to the transcription of various genes involved in cell proliferation. The RAS protein is then deactivated by the conversion of GTP to GDP by GTPase-activating proteins (GAPs). **(B)** A mutation in the *KRAS* gene produces a mutant form of RAS that prefers its active state regardless of the presence of a ligand bound to the receptor. This leads to aberrant activation of the downstream signalling, leading to constant activation of transcription factors in the nucleus. Consequently, there is unregulated transcription of various genes involved in cell growth which results in uncontrolled cell proliferation. (Created with [BioRender.com](https://biorender.com/), accessed on 14 June 2022).
