# Supplementary material for: The genetic profile and molecular subtypes of human pseudomyxoma peritonei and appendiceal mucinous neoplasms: a systematic review
Source: Cancer Metastasis Rev. 2023 Feb 1;42(1):335–59. doi: 10.1007/s10555-023-10088-0 (PMC10014681; doi:10.1007/s10555-023-10088-0)
Supplement: Supplementary file 6 — : Figure S2: GNAS in the cAMP-PKA pathway. (DOCX 662 kb) [file 10555_2023_10088_MOESM6_ESM.docx]

**Supplementary Figure S2: *GNAS* in the cAMP-PKA Pathway**

**A**


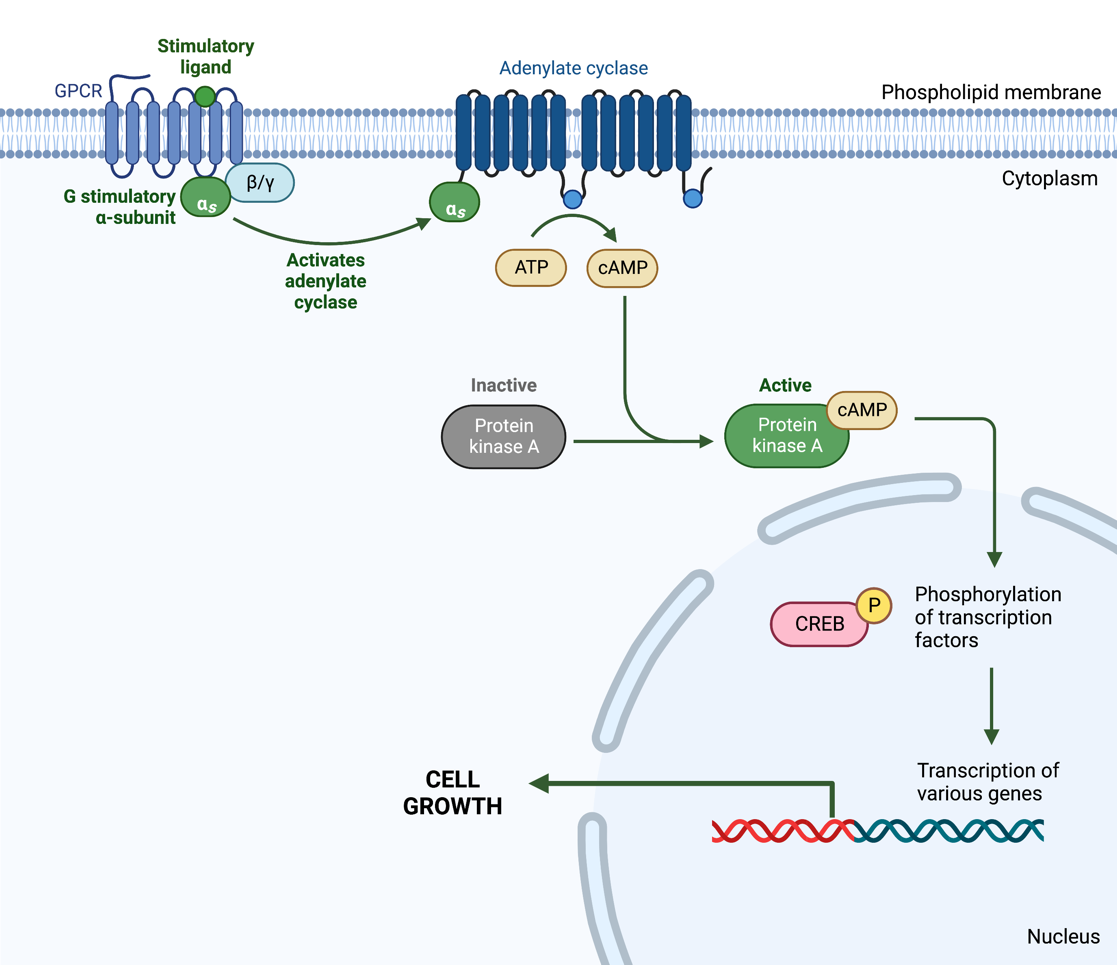


**B**


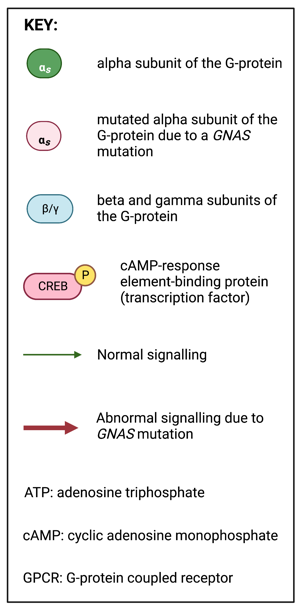

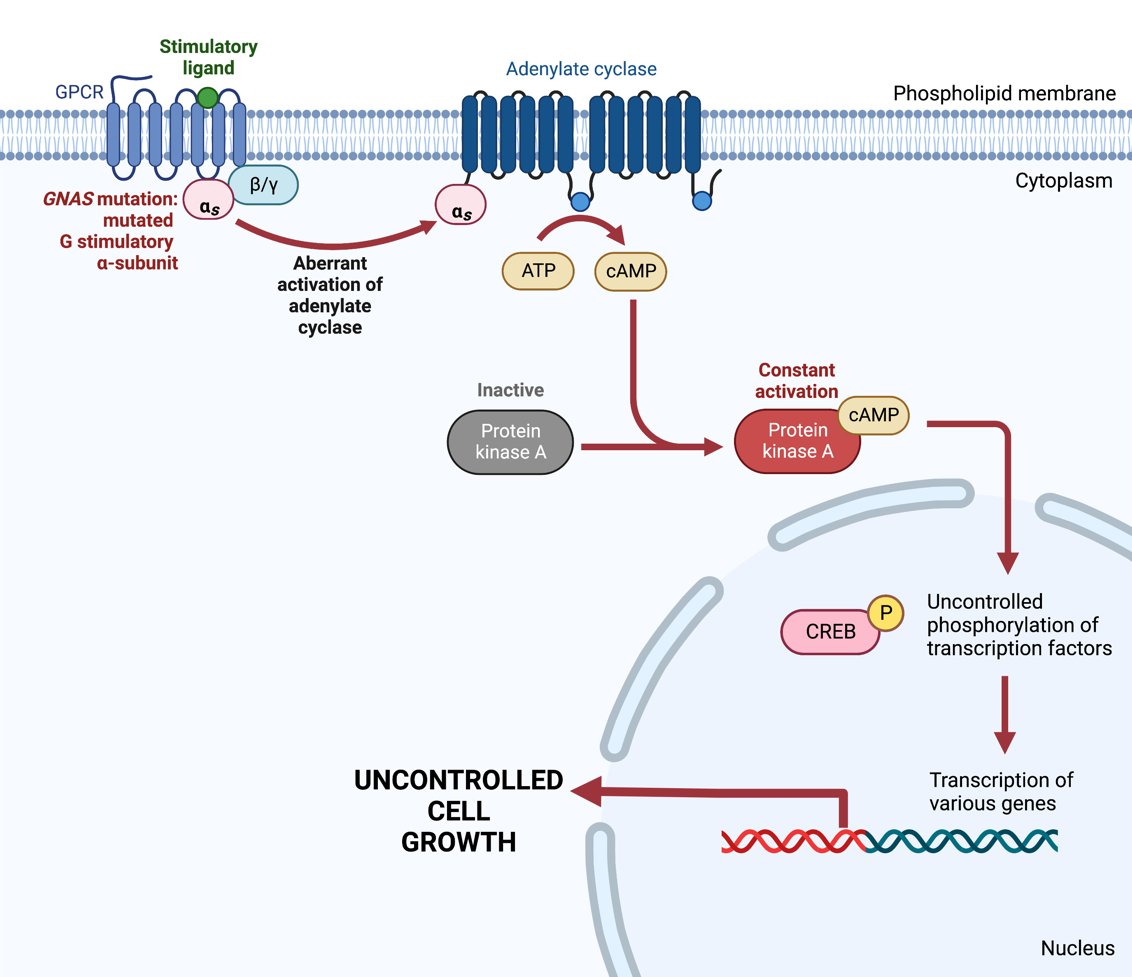


**Supplementary Figure 2**: ***GNAS* in the cAMP-PKA pathway. (A)** An extracellular ligand binds to the stimulatory part of the G-protein coupled receptor (GPCR) which activates the alpha-subunit of the G-protein. This activates adenylate cyclase which in turn activates cyclic adenosine monophosphate (cAMP), a second messenger. cAMP activates protein kinase A (PKA) which phosphorylates transcriptional factors in the nucleus. As a result, there is transcription of genes involved in cell growth. **(B)** A *GNAS* mutation codes for a mutated alpha-subunit of the G-protein which causes aberrant activation of adenylate cyclase. This leads to an increase in the downstream activation of cAMP resulting in the constant activation of PKA. Consequently, there is uncontrolled activation of transcription factors which results in the unregulated transcription of genes involved in cell growth. Based on the findings of Hannah-Shmouni et al.^81^ (Created with [BioRender.com](https://biorender.com/), accessed on 14 November 2022).
